# Large-Scale East-Asian eQTL Mapping Reveals Novel Candidate Genes for LD Mapping and the Genomic Landscape of Transcriptional Effects of Sequence Variants

**DOI:** 10.1371/journal.pone.0100924

**Published:** 2014-06-23

**Authors:** Maiko Narahara, Koichiro Higasa, Seiji Nakamura, Yasuharu Tabara, Takahisa Kawaguchi, Miho Ishii, Kenichi Matsubara, Fumihiko Matsuda, Ryo Yamada

**Affiliations:** 1 Statistical Genetics, Center for Genomic Medicine, Kyoto University Graduate School of Medicine, Kyoto, Japan; 2 Human Disease Genomics, Center for Genomic Medicine, Kyoto University Graduate School of Medicine, Kyoto, Japan; 3 DNA Chip Research Inc., Kanagawa, Japan; Ohio State University Medical Center, United States of America

## Abstract

Profiles of sequence variants that influence gene transcription are very important for understanding mechanisms that affect phenotypic variation and disease susceptibility. Using genotypes at 1.4 million SNPs and a comprehensive transcriptional profile of 15,454 coding genes and 6,113 lincRNA genes obtained from peripheral blood cells of 298 Japanese individuals, we mapped expression quantitative trait loci (eQTLs). We identified 3,804 *cis-*eQTLs (within 500 kb from target genes) and 165 *trans*-eQTLs (>500 kb away or on different chromosomes). *Cis-*eQTLs were often located in transcribed or adjacent regions of genes; among these regions, 5′ untranslated regions and 5′ flanking regions had the largest effects. Epigenetic evidence for regulatory potential accumulated in public databases explained the magnitude of the effects of our eQTLs. *Cis*-eQTLs were often located near the respective target genes, if not within genes. Large effect sizes were observed with eQTLs near target genes, and effect sizes were obviously attenuated as the eQTL distance from the gene increased. Using a very stringent significance threshold, we identified 165 large-effect *trans*-eQTLs. We used our eQTL map to assess 8,069 disease-associated SNPs identified in 1,436 genome-wide association studies (GWAS). We identified genes that might be truly causative, but GWAS might have failed to identify for 148 out of the GWAS-identified SNPs; for example, *TUFM* (*P* = 3.3E-48) was identified for inflammatory bowel disease (early onset); *ZFP90* (*P* = 4.4E-34) for ulcerative colitis; and *IDUA* (*P* = 2.2E-11) for Parkinson's disease. We identified four genes (*P*<2.0E-14) that might be related to three diseases and two hematological traits; each expression is regulated by *trans*-eQTLs on a different chromosome than the gene.

## Introduction

Variation in gene expression levels is one of the major factors causing phenotypic variation and disease susceptibility. Although gene expression levels are influenced by environmental factors, genetic variations also play an important role in transcriptional regulation; notably, about 30% of transcriptional phenotypes are heritable (*h* ^2^>30%) [1]. Additionally, many loci identified in genome-wide association studies (GWAS) are located in non-coding regions that have no known protein-coding genes, suggesting that these loci influence transcriptional regulation. Expression quantitative trait locus (eQTL) mapping is a common approach to locate genetic loci that regulate transcription, and recent development with genome-wide SNP typing arrays and gene expression microarrays has enhanced genome-wide eQTL mapping. Genome-wide eQTL maps can substantially improve our understanding of transcriptional regulation at the genetic level; they can also improve the interpretability of the results of GWAS. Moreover, comprehensive hypothesis-free scans of eQTLs can provide hypothesis-generating results; this approach may lead to the unexpected discovery of important biological phenomena. Consequently, eQTL mapping has been intensively studied in humans [1]–[9]. However, further eQTL mapping studies would be valuable because technical advances in high-throughput genome analysis are being made in terms of experiments, accumulation of knowledge, and computation. Moreover, non-coding RNAs are important regulators of gene expression, and these RNAs greatly influence many phenotypes [10], [11]. Therefore, profiling eQTLs of non-coding RNAs should be very valuable for biomedical research; however, previous eQTL studies have focused almost exclusively on protein-coding genes. Here, our study included 6,113 lincRNA probes; we identified 278 unique eQTLs that affected 326 unique lincRNA probes.

Notably, expression levels of many individual genes vary among human populations [7], [12]–[16], and this variation among populations is primarily explained by differences in genotype frequencies (*R* ^2^ of ∼0.81) among populations; nevertheless, population-specific genotypic effects may also be an important source of this variation (*R* ^2^ of ∼0.31) [13]. Additionally, low between-population replication rates of eQTLs indicate that population-specific eQTL effects exist; for example, only 37% of *cis-*eQTLs and 15% of *trans-*eQTLs identified in one population were also identified in a second population [7]. Therefore, ethnicity-specific eQTL maps may be very useful for basic and applied research. Here, we describe large-scale eQTL mapping in a Japanese population; the sample size (*n* = 298 unrelated individuals) was 3-fold larger than that in any preceding eQTL study of East Asian individuals [7], [13], [17]; moreover, updated genome and gene data were used to improve the coverage of tested transcripts over that in preceding studies. In this study, we report genome-wide, high-resolution eQTL association mapping for baseline gene expression levels in peripheral blood cells.

We identified 3,804 *cis-*eQTLs (defined as a SNP that affects expression of a gene located within 500 kb) that affected 16.9% of genes; among these *cis-*eQTLs, the mean fold difference in gene expression levels between two homozygous genotypes was 1.6-fold, and the mean proportion of transcriptional variance explained by genotype was 0.19. We also identified 165 *trans-*eQTLs (defined as a SNP that affects expression of any transcript more than 500 kb away or on a different chromosome); among these *trans-*eQTLs, the mean fold difference in gene expression levels between two homozygous genotypes was 2.1-fold, and the mean proportion of transcriptional variance explained by genotype was 0.27. *Cis-*eQTLs were more likely to be located in gene structure and the adjacent regions; specifically, 45.7% of *cis-*eQTLs were located within 1 kb of the respective differentially expressed gene (genic *cis-*eQTLs). The genic *cis-*eQTLs had a larger effect than other *cis-*eQTLs (mean |β|: 0.33 vs. 0.31, *P* = 0.00093; mean *R* ^2^ 0.21 vs. 0.17, *P* = 7.8E-11). *Cis-*eQTLs with the largest effects (top 10%) were located predominantly in genic regions (58% in genic vs. 42% in the others). Among the genic regions, 5′ untranslated regions (UTR) and upstream regions within 1 kb of a transcription start site had relatively more *cis-*eQTLs than the other regions, and *cis-*eQTLs with larger effects also tended to be located in these two types of genic regions; the mean effect size of *cis*-eQTLs in these regions were 1.4-fold larger than those of others (mean |β|: 0.45 vs. 0.32, *P* = 0.0033). The density of *cis-*eQTLs decreased exponentially with distance from respective structural genes; the majority (70%) of *cis-*eQTLs were located within 17 kb-flanking or within a target protein coding gene; and effects of individual *cis-*eQTLs became small with distance from a target gene.

eQTL analyses have been used to reliably identify variant-gene pair(s) among potential combinations of SNPs identified by GWAS and the nearby genes [18]; nevertheless, a considerable fraction of GWAS have not included eQTLs evaluation. In many GWAS, the gene closest to the significant SNP is reported as a probable causative gene. However, there are two major caveats with this practice: 1) when multiple genes are in strong linkage disequilibrium (LD) in the detected region, the reported SNP may capture an effect of a faraway gene, and thus, GWAS cannot determine which gene in the LD region is truly causative; and 2) the reported SNP may capture a transcriptional regulatory site that is located far from the regulated and causative gene. Therefore, eQTL maps may improve interpretation of GWAS results and overcome these two caveats by identifying causative genes whose expression is actually altered. We used our eQTL map to reassess 8,069 trait/disease-associated SNPs identified in 1,436 published GWAS; our eQTL map suggested different causative genes from those reported in published GWAS for 148 of the GWAS-identified SNPs.

Our eQTL mapping project is part of the Human Genetic Variation Browser (http://www.genome.med.kyoto-u.ac.jp/SnpDB/), an open-access database; this project is intended to provide researchers with integrative genomic data–including our eQTL map, summary statistics for genotypes of all SNPs used in this study, and exome sequencing data–for biomedical studies.

## Results

### Gene expression profile

Our study population comprised 298 individuals (102 male and 196 female); the mean age was 55.1 years, and age ranged from 32 to 66 years (Table S1). We treated each probe as though it represented a unique transcript, and each Entrez Gene ID represented a distinct gene. With this definition of genes, our expression profile was comprised of 30,395 autosomal transcripts (17,598 genes): 19,818 *mRNA* transcripts representing 15,454 genes, 6,113 *lincRNA* transcripts (no gene ID was assigned for any of them), and 4,464 *other* transcripts representing 3,288 genes (see Methods for classification). The numbers of genes (15,454 and 3,288) do not add up to the total (17,598) because 1,144 gene IDs were found in both mRNA and others as different transcripts. Definitions of *cis*- and *trans*-eQTLs, and local and distant SNPs are described in Methods.

### *Cis-*eQTL analysis

**Figure 1:**
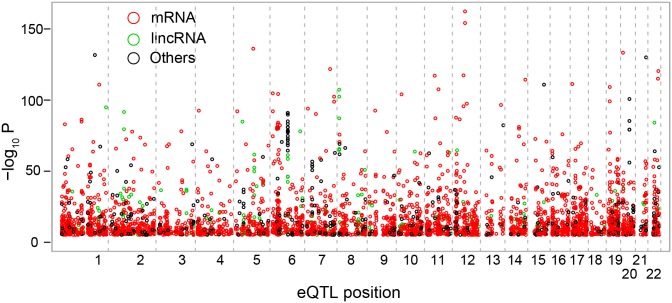
Cis-eQTL map. –log_10_ P values of cis-eQTLs are plotted against the respective chromosomal positions. eQTLs for mRNA transcripts are shown in red; lincRNA transcripts are shown in green; and other transcripts are shown in black. The vertical dashed lines separate chromosomes.

A distribution of *P* values for all local SNP-transcript pairs showed an excess of small *P* values (Figure S1A), suggesting that a substantial fraction of associations are truly positive. With the false discovery rate (FDR) <5%, we identified 3,804 *cis*-eQTLs transcript pairs (Figure 1, Table 1). 12.5%, or 16.9%, of all tested transcripts, or genes, were *cis*-regulated (Table 1). The complete list of the *cis*-eQTLs with annotation and statistics is provided in File S1.

**Table 1:** Summary statistics and counts of *cis*- and *trans*-eQTLs at thresholds by *R2* or |β|.

		*cis-*eQTL(*n* = 3,804 by FDR <5%)				*trans-*eQTL(*n* = 165 by FWER <5%)			
		All	mRNA	lincRNA	Other	All	mRNA	lincRNA	Other
**#eQTLs-transcript pairs**		3,804	2,995	293	516	165	91	49	25
**#unique eQTLs**		3,385	2,779	244	440	105	65	34	21
**#unique transcripts (%)**		3,804 (12.5%)	2,995 (15.1%)	293 (4.8%)	516 (11.6%)	114 (0.4%)	60 (0.3%)	34 (0.6%)	20 (0.4%)
**#unique genes (%)**		2,973 (16.9%)	2,667 (17.3%)	0	357 (10.9%)	74 (0.4%)	57 (0.4%)	0	17 (0.5%)
**#unique transcripts without gene ID**		455	28	293	134	54	1	49	4
***R*** **^2^ mean±SD**		0.19±0.15	0.19±0.15	0.20±0.16	0.21±0.18	0.27±0.12	0.27±0.12	0.29±0.13	0.23±0.07
***R*** **^2^ median**±**IQR**		0.13±0.15	0.13±0.14	0.13±0.15	0.15±0.17	0.23±0.12	0.23±0.12	0.26±0.11	0.21±0.09
**|β| mean**±**SD**		0.33±0.33	0.32±0.32	0.38±0.33	0.38±0.34	0.53±0.35	0.50±0.38	0.59±0.31	0.51±0.33
**|β| median**±**IQR**		0.24±0.24	0.23±0.23	0.30±0.31	0.28±0.25	0.47±0.41	0.40±0.44	0.58±0.34	0.43±0.34
**Stat.**	**Cutoff**								
***R*** **^2^**	**0.1**	2,568	2,022	192	354	165	91	49	25
	**0.3**	665	500	56	109	45	25	15	5
	**0.5**	245	171	22	52	10	5	5	0
	**0.7**	67	43	8	16	2	2	0	0
	**0.9**	2	2	0	0	0	0	0	0
**|β|**	**0.3**	1,440	1,053	146	241	118	60	40	18
	**0.9**	155	122	11	22	23	13	6	4
	**1.5**	55	44	3	8	3	1	1	1
	**2.1**	26	19	2	5	1	1	0	0
	**2.7**	12	9	1	2	1	1	0	0

FDR: false discovery rate; FWER: family-wise error rate; SD: standard deviation; IQR: inter-quartile range; *R* ^2^: proportion of phenotypic variances explained by genotypes; |β|: absolute value of coefficient of genotypes.

The sum of #unique eQTLs counted within RNA types is not necessarily equal to #unique eQTLs counted for all transcripts because the same eQTLs may be counted in more than one RNA types. The number of genes for All and each type do not match for a similar reason.

**Figure 2:**
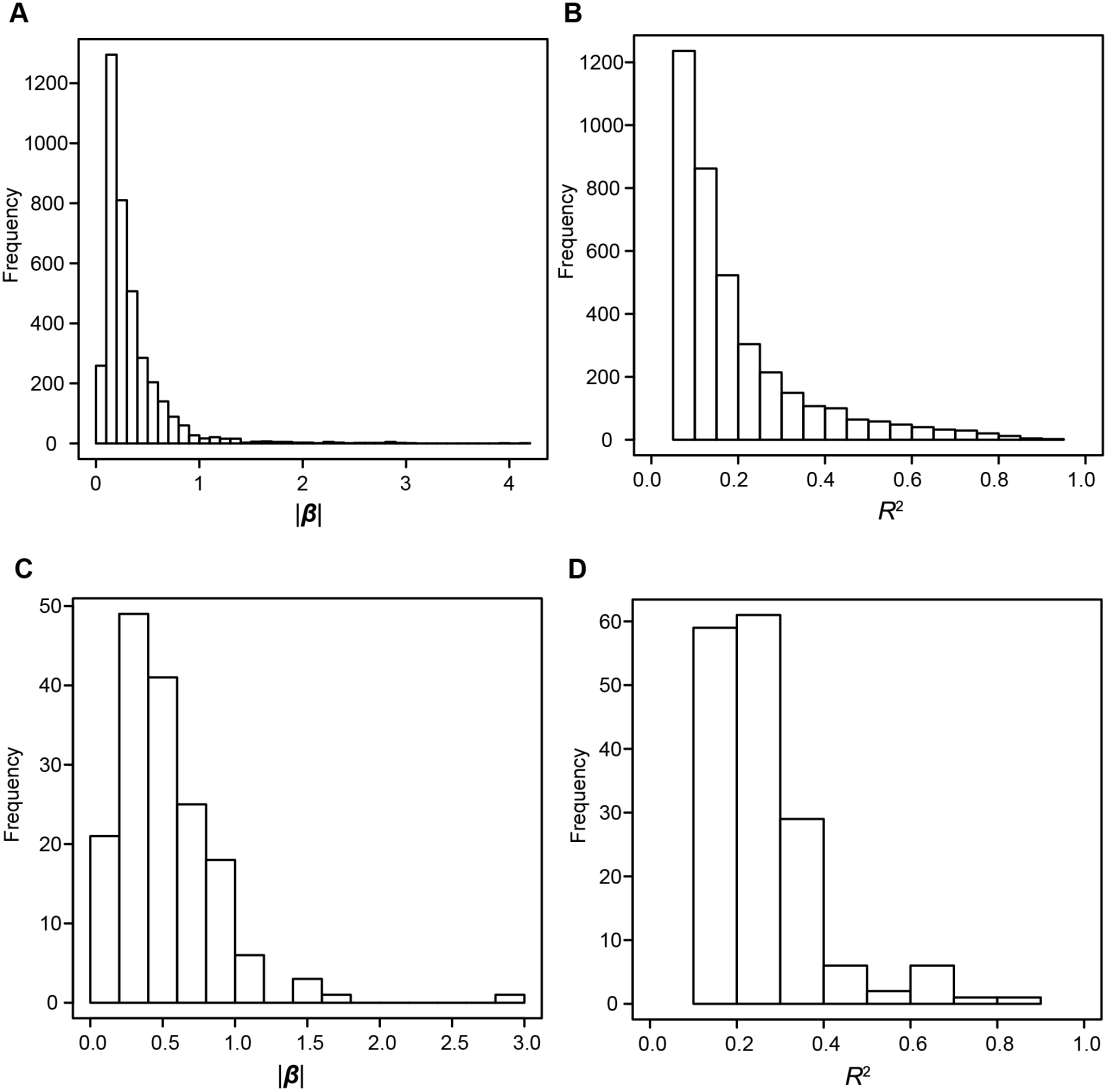
Histograms of effects of eQTLs. A, B) Histograms of |β| values (A) and of *R* ^2^ values (B) of *cis-*eQTLs are shown. C, D) Histograms of |β| values (C) and *R* ^2^ values (D) of *trans-*eQTLs are shown.

We used two statistics as measures for magnitudes of effects of eQTLs; the coefficient of genotypes was designated β, or its absolute value |β|, and the proportion of transcriptional variance explained by genotypes was designated *R* ^2^ (see supplementary note in File S3 for more explanation). *Cis-*eQTLs with large effects were abundant (Figure 2A, 2B and Table 1): for example, the number of *cis-*eQTLs with |β| values larger than 0.3, which corresponds to a 1.5-fold change between two homozygous genotypes, was 1,440 (4.7%) of all tested transcripts. The numbers of *cis-*eQTLs with *R* ^2^ values larger than 0.1 were 2,568 (8.4%) of all examined transcripts.

#### Gene-based functional categories and protein consequences

**Figure 3:**
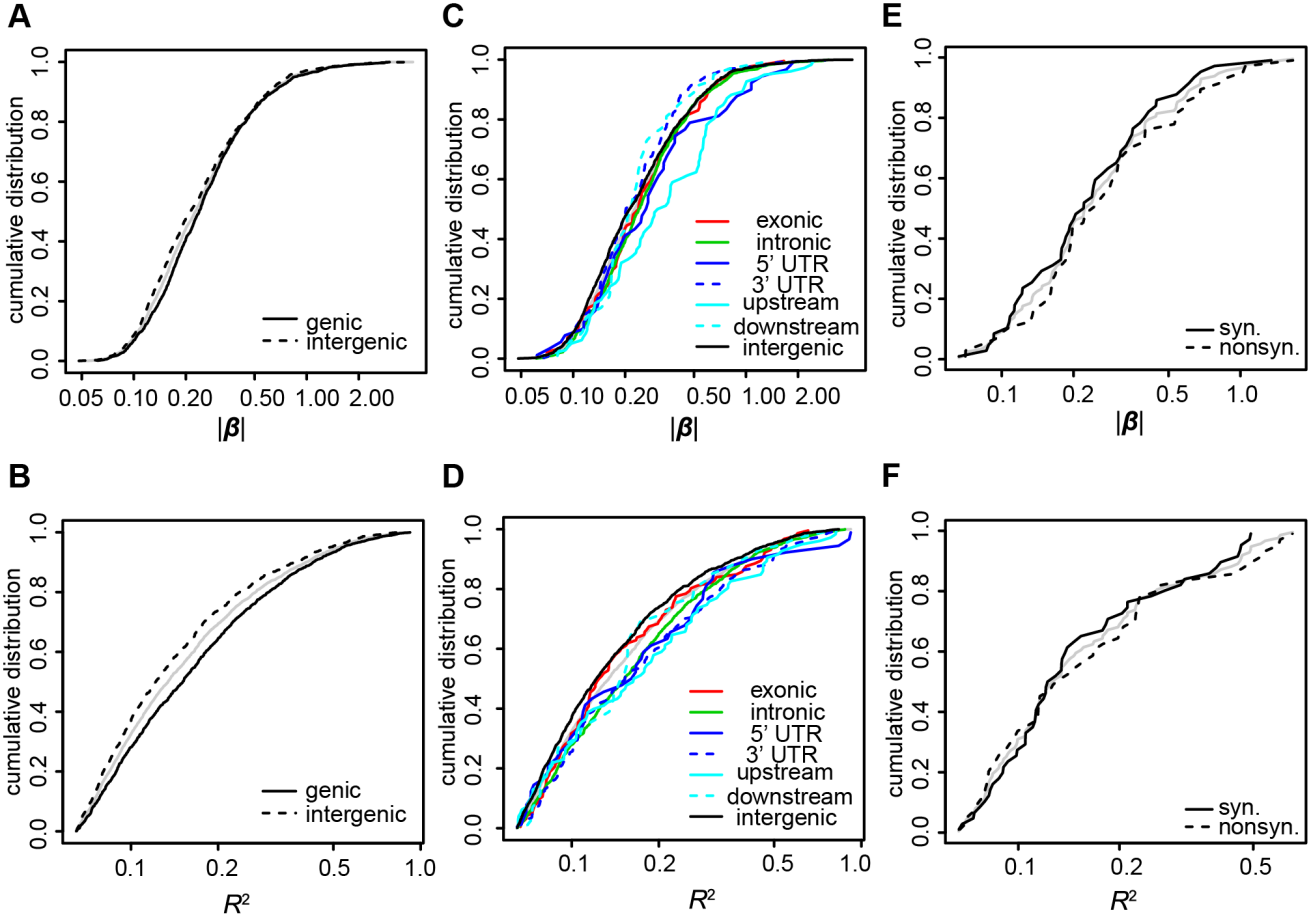
Cumulative curves of effect magnitudes of *cis-*eQTLs in gene-structure-based functional categories. Cumulative curves represent the distributions of |β| values or *R* ^2^ values of *cis*-eQTLs in each category. Cumulative distribution of all *cis-*eQTLs (A–D) or all exonic *cis-*eQTLs (E–F) are shown in grey. The X axis is a log scale. A, B) Distributions of genic and intergenic *cis-*eQTLs for |β| values (A) or for *R* ^2^ values (B). C, D) Distributions of genic subcategories and intergenics for |β| values (C) or for *R* ^2^ values (D). E, F) Distributions of nonsynonymous and synonymous eQTLs for |β| values (E) and for *R* ^2^ values (F).

Next, we analyzed the *cis-*eQTLs in terms of gene-based functional categories of SNPs. Here, we analyzed the *cis-*eQTLs that affected mRNAs because the structures of the coding genes represented by these transcripts were the most clearly annotated. First, we compared SNPs in genic regions, those within genes and 1 kb upstream or downstream of genes, with SNPs in intergenic regions. We define *enrichment* as the fold change in proportion that each group constitutes among *cis*-eQTLs compared to among all local SNPs. The enrichment of genic SNPs was 7.04 (45.74% of *cis*-eQTLs vs. 6.50% of all local SNPs, Table 2). Moreover, *cis-*eQTLs had significantly stronger effects than did intergenic *cis*-eQTLs (mean |β| values 0.33 vs. 0.31, *P* = 0.00093; mean *R* ^2^ values 0.21 vs. 0.17, *P* = 7.8E-11, Table 2, Figure 3A, 3B). To further characterize genic eQTLs, we compared genic subcategories **(**exonic excluding UTRs, intronic, 5′ UTR, 3′ UTR, upstream, and downstream). Upstream and 5′ UTR were distinctly important compared to other genic subcategories: The most intense enrichment was observed for the 5′ UTR (41.79 fold), followed by upstream (27.95 fold), and these two subcategories had largest mean |β| and *R* ^2^ values (Table 2). Mean |β| and *R* ^2^ values of seven categories (six genic subcategories and the intergenic category) were significantly different (ANOVA *P* = 1.1E-05 for |β| and *P* = 2.5E-08 for *R* ^2^). Significantly different category pairs are shown in Table S2: The upstream had significantly larger |β| value than intron, 3′ UTR, or downstream, and for *R* ^2^ values, no pairs of genic subcategories were significantly different. Consistently, *cis-*eQTLs with larger |β| values were more common in 5′ UTR or upstream regions than in other regions (Figure 3C); additionally, *R* ^2^ value of each genic subcategories tended to be larger than the *R* ^2^ value of the intergenic category (Figure 3D). We did not observe statistically significant difference between nonsynonymous and synonymous SNPs in enrichment or mean effect sizes (eQTL enrichment: Fisher's exact *P* = 0.56, |β|: *P* = 0.257 *R* ^2^: *P* = 0.70), and the distribution of |β| values or *R* ^2^ values was similar (Figure 3E, 3F).

**Table 2:** Counts and proportions of gene structure-based categories and protein consequences in local SNPs and *cis-*eQTLs.

					Mean effect	
	Categories	Local SNPs (%)	*cis*-eQTLs (%)	Enrich	β	*R* ^2^
Intergenic		10,268,814 (93.11)	1,523 (50.85)	0.55	0.31	0.17
Genic		716,576 (6.50)	1,370 (45.74)	7.04	0.33	0.21
	Exonic	25,822 (0.23)	109 (3.64)	15.54	0.32	0.19
	Splicing	28 (0.00)	0 (0.00)	-	-	-
	Intronic	633,398 (5.74)	889 (29.68)	5.17	0.33	0.20
	3′ UTR	29,609 (0.27)	188 (6.28)	23.38	0.28	0.22
	5′ UTR	3,965 (0.04)	45 (1.50)	41.79	0.41	0.22
	Upstream	11,727 (0.11)	89 (2.97)	27.95	0.47	0.23
	Downstream	12,027 (0.11)	50 (1.67)	15.31	0.27	0.19
N.A.		42,870 (0.39)	102 (3.41)	8.76	0.38	0.21
**Total**		**11,028,260 (100.00)**	**2,995 (100.00)**	**1.00**	**0.32**	**0.19**
Exonic	nonsyn	11,739 (45.46)	52 (47.71)	1.05	0.35	0.20
	syn	13,662 (52.91)	53 (48.62)	0.92	0.29	0.18
	stopgain	52 (0.20)	2 (1.83)	9.11	0.45	0.24
	stoploss	10 (0.04)	0 (0.00)	-	-	-
	N.A.	359 (1.39)	2 (1.83)	1.32	0.32	0.19
**Total**		**25,822 (100.00)**	**109 (100.00)**	**1.00**	**0.32**	**0.19**

Local SNPs and *cis-*eQTLs that affect mRNA transcripts are counted within each gene-based functional category (upper panel) and for each protein consequence (lower panel).

Enrich: the fold change in proportion that each group constitutes among *cis*-eQTLs compared to among all local SNPs.

The category “Exonic” does not include 5′ and 3′ untranslated regions (UTRs); “Upstream” and “Downstream” each includes regions within 1 kb from transcription start or end sites of genes, respectively; “Splicing” includes SNPs 2 bp from exon-intron splicing junctions and within an intron; SNPs 2 bp from a splice junction and within an exon are designated “Exonic”); “Intronic” includes SNPs in introns, but not those 2 bp from exon-intron splicing junctions; “nonsyn” indicates a SNP in an Exonic that is non-synonymous; “syn” indicates an SNP in an Exonic that is synonymous; “stopgain” indicates an SNP in an Exonic and with a variant that causes the creation of stop codon; “stoploss” indicates an SNP in an Exonic and with a variant that eliminates a stop codon.

N.A. means “Not Available” and includes SNPs that were found in a gene, but that could not be assigned to a specific functional category.

Totals for gene-structure-based classification and protein consequences are shown in bold font.

#### Intergenic eQTLs and RegulomeDB class

**Figure 4:**
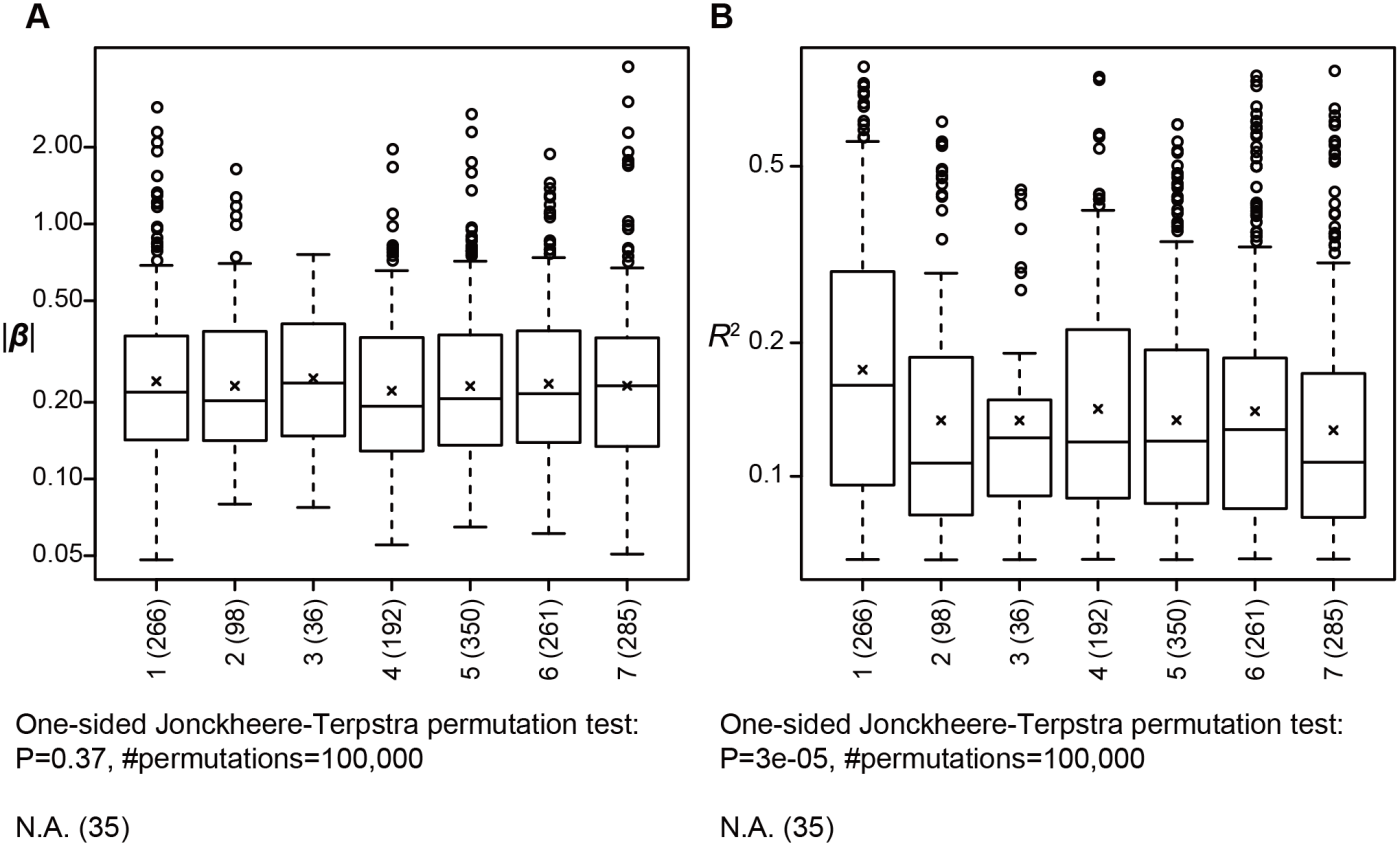
Trend in effects associated with regulatory classes of intergenic *cis-*eQTLs. The box-and-whisker plots show distributions of |β| values (A) or of *R* ^2^ values (B) of intergenic *cis*-eQTLs that affect mRNA transcripts for regulatory classes defined by the RegulomeDB. A cross indicates the mean effect of each class. The number of *cis*-eQTLs belonging to each class is shown in the parentheses following the class name. Jonckheere-Terpstra permutation test was used to test each trend, and the results are shown under the box-and-whisker plots. N.A.: not available.

Next, we characterized the intergenic *cis-*eQTLs; again, we focused only on *cis-*eQTLs that affected mRNA transcripts. Although the mean effects of intergenic eQTLs were significantly smaller than those of genic eQTLs, intergenic eQTLs are still important because the 1,523 intergenic eQTLs constituted 50.85% of the *cis-*eQTLs (Table 2). Therefore, to further characterize this large number of intergenic eQTLs we analyzed each in terms of regulatory potential; this potential was predicted based on known epigenetic evidence. We classified each intergenic eQTL into one of seven numbered categories based on the RegulomeDB, which indicates how likely a variant is to disrupt transcription factor binding [19] (see Methods for the classification). We observed statistically significant trends in means of *R* ^2^ values (*P* = 3e-05) but not in means of |β| (*P* = 0.37); the eQTLs classified into higher potential classes had stronger effects (Figure 4). Although the eQTLs in Category 1 had the largest mean *R* ^2^, the means of other categories were not apparently different.

#### Relationship between eQTL effects and distance

**Figure 5:**
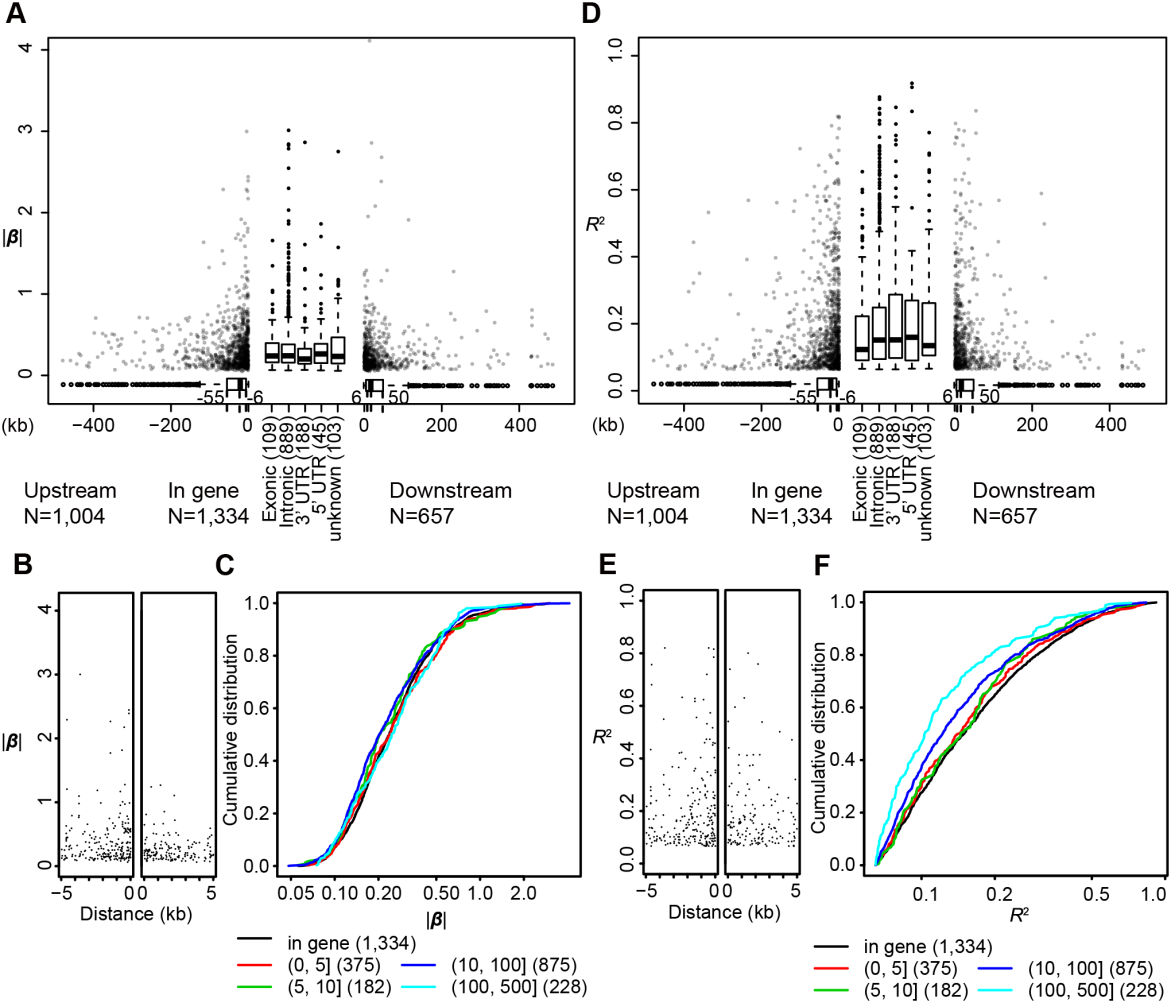
Relationships between effects of *cis-*eQTLs and distance from genes. |β| values (A) and *R* ^2^ values (D) of *cis-*eQTLs that affect mRNA transcripts are plotted against distances from the respective target genes by scatter (non-transcribed regions) and by box-and-whisker plots (transcribed regions). eQTLs in transcribed regions are shown for each gene-structure-based category. The number following each category name represents the number of *cis*-eQTLs classified into that category. Negative distance values indicate that the eQTL is upstream of the target gene, and positive values indicate that it is downstream, regarding transcriptional directions. Distributions of distances are represented by box-and-whisker plots below the scatters. Magnified view for <5 kb of genes is shown for |β| (B) and *R* ^2^ (E). Cumulative distribution of |β| (C) and *R* ^2^ (F) of eQTLs are shown for each division of eQTLs; each division represent a defined distance (kb) from the respective target gene. The number in the parentheses following each distance range in the legend is the number of *cis*-eQTLs identified in that range. The X-axis is a log scale. One eQTL located within a gene (*C16orf55*) that was assigned function of “downstream” is shown as “unknown”; therefore, the number of “In gene” eQTLs shown in (A) and (D) is the sum of the numbers of Exonic, Splicing, Intronic, 5′ UTR, 3′ UTR, and N.A. in Table 2 plus 1.

Next, we investigated whether and how distances between *cis-*eQTLs and their mRNA transcripts were related to the magnitudes of the effects. Distances and effects were strongly correlated with exponential decay in both the 5′ and 3′ directions (Figure 5A, 5D). eQTLs were concentrated in regions near genes; 73% of *cis-*eQTLs outside genes were located within 50 kb of their target genes. Promoters are usually located within 100 bp upstream of genes; nevertheless, eQTLs were not apparently enriched in promoter regions (Figure 5B, 5E). As distances increased, eQTLs of small effect became more common; this trend is evident in *R* ^2^ values for eQTLs>100 kb, but not in the |β| values (Figure 5C, 5F).

### *Trans-*eQTL analysis

*P* values of all tests for distant SNPs were distributed almost uniformly with a slight excess of small *P* values, suggesting that only a small fraction of distant SNPs affected transcriptional regulation (Figure S1B). With a stringent multiple-testing correction (*P*<1.15E-12) and excluding redundancy due to LD and excluding possible false positives because of cross-hybridization to local regions (see Methods), we identified 165 combinations of independent *trans-*eQTLs and transcripts that comprised 114 unique transcripts (74 genes) affected by 105 unique *trans-*eQTLs, and these *trans-*regulated transcripts represented 0.4% of all tested transcripts (Table 1). Large *trans-*effects were identified (Figure 2C, 2D and Table 1). The number of *trans-*eQTLs with |β| values larger than 0.3 was 118 for all transcripts, which covers 0.39% of all tested transcripts; additionally, each *trans-*eQTL had an *R* ^2^ value larger than 0.1 (Table 1). The ratio of the number of *trans-*eQTLs to the number of *cis*-eQTLs at the same cutoff values of |β| or *R* ^2^ tend to be smaller as the cutoff value became larger (Table 1), indicating strong effects are more abundant in *cis*-eQTLs. All the 165 *trans-*eQTL transcript pairs are provided in File S1.

We assigned RegulomeDB classes [19] to *trans*-eQTLs, and tested a trend in the same manner as *cis*-eQTLs. Unlike *cis*-eQTLs, we did not observe statistically significant trends in means of *R* ^2^ values (P = 0.99) or in means of |β| (P = 0.92).

### Multi-regulatory eQTLs

A *cis-*eQTL that is associated with expression of multiple genes might indicate the existence of a long-range enhancer/repressor that influences the expression of a cluster of genes in a region. We identified 6 *cis-*eQTLs that were each associated with expression levels of three or more mRNA-coding genes (Table 3). These multi-regulatory *cis*-eQTLs were each associated with the regulated transcripts in the same direction (Figure S4A).

**Table 3:** Multi-regulatory *cis*-eQTLs and *trans-*eQTLs.

					LD block			
eQTL	Chr	Position	MAF	HWE-P	Start	End	Length	Gene Symbol
***cis***								
rs7522860	1	156,275,281	0.49	0.644	156,208,230	156,314,627	106,398	*TMEM79;SMG5;C1orf85;PAQR6*
rs6464103	7	150,478,385	0.37	0.711	150,476,888	150,478,385	1,498	*TMEM176B;TMEM176A;ABP1*
rs4390300	10	60,144,207	0.47	0.817	60,144,207	60,168,003	23,797	*IPMK;UBE2D1;TFAM*
rs2416549	12	11,325,804	0.24	0.116	11,045,512	11,349,454	303,943	*TAS2R14;TAS2R30;PRB1*
rs35969491	12	11,339,020	0.24	0.084	11,045,512	11,349,454	303,943	*TAS2R10;PRR4;PRH2;PRB4*
rs7226263	17	44,814,884	0.32	0.111	44,788,310	44,853,872	65,563	*WNT3;ARL17B;ARL17A;NSF*
***trans***								
rs116711766	1	160,093,165	0.075	0.3909	160,093,165	160,093,165	1	*ITGA7;MC1R;FAM22G*
rs11718621	3	40,362,122	0.288	1.0000	40,362,122	40,463,063	100,942	*DIRC1;MAB21L2;PRSS36;* *HIST2H2BF;KRTAP19–2;FSD1;LRRD1*
rs6773917	3	40,469,254	0.492	0.4881	40,373,259	40,498,845	125,587	*DIRC1;MAB21L2;PRSS36;* *HIST2H2BF;NEURL;KRTAP19–2;FSD1;LRRD1*
rs7801498	7	102,089,595	0.368	0.8039	102,089,595	102,089,595	1	*MUC4;GFRA1;MIOX;GYPA*
rs10873415	14	92,558,171	0.380	0.0097	92,434,957	92,558,171	123,215	*GADD45GIP1;SOX13;TFEB;EIF2C1*

Chr, Position: chromosomal positions of eQTLs; MAF: minor allele frequency; HWE-P: Hardy-Weinberg Equilibrium test *P* value; LD block: range in which SNPs in LD (*r* ^2^>0.8) with the eQTLs exist.

A *trans*-eQTL that is associated with the expression of multiple genes is a potential master regulator. Our *trans*-eQTL map indicates that there are some *trans-*eQTL hotspots that were involved in multiple genes across the genome (Figure S5). We identified 5 *trans-*eQTLs that were each associated with three or more mRNA-coding genes (Table 3). Rs7801498 was also identified as a *cis*-eQTL for two genes (*LRWD1* and *ORAI2*). Notably, again, these multi-regulatory *trans-*eQTLs were each associated with the regulated transcripts in the same direction (Figure S4B).

### Replication analysis with independent studies

We compared our eQTLs to a meta-analysis of eQTL studies of whole blood samples conducted by Westra *et al.* [20]. They analyzed samples from 5,311 individuals from European populations. We focused on 15,733 genes that were commonly tested in both studies. At FDR<0.05, 10.6% of the genes were found *cis*-regulated in both studies; 60.9% of 2,750 *cis*-regulated genes in this study were replicated; and the concordance rate (*i.e.*, consistently *cis*-regulated or non-*cis*-regulated in both studies) was 68.8%. The concordance rate increased as FDR thresholds became more stringent up to 74.4% at FDR<1E-06. The replication rate of our *cis*-regulated genes was significantly associated with median non-adjusted expression levels (logistic regression *P*<2E-16, log OR = 0.14), but not with SD (*P* = 0.14). 45.2% of 3,106 pairs of our *cis*-eQTLs (including SNPs in *r* ^2^>0.8) and genes tested in the meta-analysis were replicated. For replication of *trans-*eQTLs we found 978 distant SNP-transcript pairs in our results that corresponded to *trans*-eQTL-gene pairs identified in the meta-analysis. Six pairs were significant at *P*<5.1E-05, which corresponds to Bonferroni-corrected *P* = 0.05 for 978 tests (Table S3). Particularly, *trans*-eQTL for *CALD1* was replicated at the original significance level (*P* = 5.30E-16). Regarding that only the limited number of SNP-gene pairs were tested in common with the meta-analysis for *trans*-eQTLs, we also compared our *trans-*eQTLs with those identified for whole blood samples obtained from 76 Japanese individuals [17]. Over 8.6 billion tests for SNP-gene pairs were performed in both studies. Of the common tests, 41 and 2 pairs were identified as *trans-*eQTLs in the current and previous studies, respectively. We identified 1 *trans-*eQTL-gene pair exactly consistent between the studies (rs4487686 for *POLR2J4*). Regarding the number of performed tests, identifying one consistent result by chance is extremely unlikely (Fisher's exact test *P*<5E-08).

### Application of the eQTL map to interpretation of GWAS results

eQTL maps improve interpretation of GWAS results by linking SNPs and genes whose expressions are actually altered. We used previously published GWAS of Crohn's disease to comprehensively illustrate how our eQTL map improves interpretation of GWAS results. We identified 12 records for which our eQTL maps were informative for interpretation among all 220 records for Crohn's disease obtained from the NHRGI GWAS Catalog (http://www.genome.gov/gwastudies/) (Table 4). We define the following four informative cases for results of applying our eQTL map to GWAS results; a GWAS result is classified into Case 1 when the eQTL map may suggest different possible interpretation for GWAS, Case 2 when the eQTL map supports the interpretation provided by GWAS, Case 3 when the eQTL map helped to prioritize multiple genes inconclusively reported by the GWAS, or Case 4 when a *trans-*effect of GWAS-identified SNP was suggested (see supplementary note in File S3 for detailed definition).

**Table 4:** Summary of GWAS records associated with Crohn's disease and eQTL mapping results.

		Suggested genes		SNPs			eQTL statistics			Top local SNP for GWAS gene			
Case	Record	GWAS	eQTL	GWAS	eQTL	*r* ^2^	β	*P*	*P_c_*	SNP	β	*P*	*r* ^2^
Case 1	1[21]	*CCR6* a	*RNASET2*	rs415890	rs400837	0.99	−0.36	2.7E-39	0.87	Not tested			
	2[21]	*FADS1*	*FADS2*	rs102275	rs108499	0.97	0.16	3.2E-10	0.74	rs174570	0.17	6.2E-07	0.99
	3[21]	*PRDX5*	*CCDC88B*	rs694739	rs600377	0.85	−0.26	1.0E-06	0.51	rs2286614	0.42	4.5E-23	0.01
		*ESRRA*								rs641811	0.06	n.s.	0.01
	4[21]	*IKZF3*	*GSDMB*	rs2872507	rs1008723	0.98	−0.38	6.9E-38	0.81	rs56030650	0.05	n.s.	0.01
		*ZPBP2*								rs62065216	–0.09	n.s.	0.01
		*ORMDL3*								rs1054609	–0.18	3.6E-14	0.98
		*GSMDL* a								Not tested			
	5[22]	*ORMDL3*	*GSDMB*	rs2872507	rs1008723	0.98	−0.38	6.9E-38	0.81	rs1054609	–0.18	3.6E-14	0.98
	6[21]	*RTEL1*	*ZGPAT*	rs4809330	rs6011058	1.00	0.09	2.9E-07	1.00	rs2252258	–0.05	n.s.	0.002
		*SLC2A4RG*								rs310609	–0.07	n.s.	0.02
		*TNFRS-F6B* a								Not tested			
Case 2	7[21]	*PLCL1*	*PLCL1*	rs6738825	rs1866664	0.98	−0.25	3.0E-07	0.81	rs1866664	–0.25	3.0E-07	1
	8[21]	*IRGM*	*IRGM*	rs7714584	rs1428554	1.00	−0.40	3.4E-13	0.98	rs1428554	–0.40	3.4E-13	1
	9[22]	*IRGM*	*IRGM*	rs11747270	rs1428554	1.00	−0.40	3.4E-13	0.98	rs1428554	–0.40	3.4E-13	1
	10[23]	*IRGM*	*IRGM*	rs13361189	rs1428554	1.00	−0.40	3.4E-13	0.98	rs1428554	–0.40	3.4E-13	1
Case 3	11[21]	*ITLN1* b	*ITLN1*	rs4656940	rs11265498	1.00	−0.67	2.4E-17	1.00	rs11265498	–0.67	2.4E-17	1
		*CD244*								rs574610	–0.12	n.s.	0.16
	12[46]	*RNASET2* b	*RNASET2*	rs2149085	rs400837	0.99	−0.36	2.7E-39	0.87	rs400837	–0.36	2.7E-39	1
		*FGFR1OP*								rs73039162	0.68	7.5E-45	0.078
		*CCR6* a								Not tested			
		*MIR3939* a								Not tested			

a The GWAS-reported gene was not included in our study.

b GWAS-reported genes that match the eQTL-suggested genes in Case 3.

*r* ^2^: correlation of genotypes for linkage disequilibrium between the GWAS-identified SNP and *cis*-eQTL (in the “SNPs” column), or between the top local SNP for GWAS gene and *cis*-eQTL (in the “Top local SNP for GWAS Gene” column).

*P_c_*: *P* value of a conditional regression on genotypes of GWAS-identified SNP.

Genes suggested by GWAS and our eQTL map are listed in the “Suggested genes” column; eQTL statistics are listed in the “eQTL statistics” column; most significant local SNP for the GWAS-reported gene is shown in the “Top local SNP for GWAS gene” column.

n.s: not significant.

For an example of Case 1, an intergenic SNP, rs694739, was identified in a GWAS of Crohn's disease (record 3 in Table 4); the study reported *PRDX5* and *ESRRA* as putative causative genes [21]. The GWAS-identified SNP was found in LD (*r* ^2^ = 0.85) with a *cis*-eQTL (rs600377) for *CCDC88B* in our eQTL map (β = –0.26, *P* = 1.0E-06). A *cis*-eQTL was identified for *PRDX5*, but the *cis-*eQTLs for *PRDX5* and *CCDC88B* were not in LD (*r* ^2^ = 0.01); and after correcting for the genotypes of the GWAS-identified SNP, the *cis*-effect on *CCDC88B* expression was not significant (*P_c_* = 0.51). Therefore, given the eQTL map, the most likely causative gene was *CCDC88B.* Based on our analyses, 6 of the 12 records were classified into Case 1, and thus, in each of these records, the eQTL-suggested gene should also be considered as another candidate gene.

Four of the 12 records were classified into Case 2. Three intergenic SNPs (rs7714584, rs11747270, rs13361189) were each reported in GWAS [21]–[23]; and in each study, *IRGM* was suggested as the candidate gene (records 8–10 in Table 4). All of these SNPs were each in perfect LD (*r* ^2^ = 1.00) with a *cis*-eQTL (rs1428554) that influenced expressions of *IRGM* (β = –0.40, *P* = 3.4E-13). None of the three SNPs were in LD (*r* ^2^>0.8) with any other *cis*-eQTLs that affected any other gene. Therefore, our eQTL analysis supported the conclusions of the GWAS.

As an example of Case 3, a GWAS (record 11 in Table 4) identified rs4656940 (in the intron of *CD244*) reporting two candidate causative genes (*CD244 and ITLN1*) [21]. The reported SNP was in perfect LD with a *cis*-eQTL (rs11265498) that influenced expressions of *ITLN1* (β = −0.67, *P* = 2.4E-17), where no *cis*-eQTL was identified for *CD244*. Therefore, our eQTL map indicated that *ITLN1* was the most likely causative gene. Our eQTL map helped to prioritize candidate genes for two of 12 records.

Any records for Crohn's disease were not classified into Case 4. In all GWAS records, we identified 13 Case-4 records (File S2). For instance, rs1354034 (in the intron of *ARHGEF3*, on chr3) was reportedly associated with platelet counts and mean platelet volume [24], [25], and *ARHGEF3* was identified as a putative causative gene. In our eQTL map, the reported SNP was not associated with the expression of *ARHGEF3* or any other tested gene on the same chromosome, but with *CALD1* on a different chromosome, chr7 (β = –0.48, *P* = 5.3E-16). For another example, rs2517713 (intergenic, on chr6) was identified in a study of nasopharyngeal carcinoma [26] and *HLA-A* was reported as a putative causative gene. In our eQTL map, the reported SNP was not associated with the expression of *HLA-A* or any other tested gene on the same chromosome, but of *NRSN2* on a different chromosome, chr20 (β = –0.21, *P* = 2.2E-15). Notably, decreased expression of *NRSN2* was reported to be associated with hepatocellular carcinoma [27].

Similarly, we analyzed 10,076 (8,069) GWAS records (unique SNPs). We identified 386 cases in which *cis*- or *trans*-effects were identified for the reported SNPs, and classified each into one of the four cases; we found 191 (148) Case-1 records, 97 (80) Case-2 records, 85 (60) Case-3 records, and 13 (6) Case-4 records. We identified 6 lincRNAs in the Case-1 records that were most significantly associated with GWAS-reported SNPs. In summary, our eQTL map was informative for 3.8% of the GWAS records, each of which was classified into one of the four cases; 1.9% into Case 1, 1.0% into Case 2, 0.8% into Case 3, and 0.1% into Case 4. We provide the results of our application of our eQTL map to the GWAS records in File S2.

## Discussion

This study identified the largest number of eQTLs for East Asian whole blood samples to our knowledge. We identified 3,804 *cis-*eQTLs and 165 *trans-*eQTLs. *Cis-*effects were previously found for 44% (6,418 genes) of tested genes [20] for Caucasian whole blood samples. In the current study, *cis*-effects were found for 16.9% of the tested genes, which is in line with estimated powers in a previous study [28].

We identified 74 genes with *trans-*effects, which constituted 0.4% of tested genes. We believe that we underestimated the proportion of true *trans-*effects because we used the most stringent corrections for multiple testing. In fact, the smallest *R* ^2^ for any of the *trans-*eQTLs (*R* ^2^ = 0.16) was 2.4-fold greater than the smallest *R* ^2^ for any identified *cis-*eQTLs (*R* ^2^ = 0.065).

We analyzed and characterized our eQTLs in various aspects; 1) *cis-*eQTLs in terms of gene structure, epigenetic factors, and distance from genes; 2) multi-regulatory eQTLs; 3) eQTLs for mRNA as compared to those for lincRNAs; 4) application of eQTL maps to GWAS results; and 5) replication with independent samples.

### 1) *Cis-*eQTL analyses

The comparison between the genic and intergenic *cis*-eQTLs suggested that factors involved in expression levels are more enriched and stronger in genic regions (those located within a gene or within 1 kb of a gene) than intergenic regions (>1 kb from genes). All genic subcategories were each overrepresented compared to the intergenic regions (Table 2). We also showed that upstream and 5′-UTR regions particularly had strong effects compared to other genic regions. It would be reasonable to consider that upstream regions are important because transcription factor binding sites and transcription regulatory modules are enriched in 5′ flanking regions of genes. Strong effects in 5′ UTRs would imply that post-transcriptional regulation via 5′ UTRs has a particularly strong impact on expression levels. The significant association between *R* ^2^ of *cis-*eQTLs and epigenetic classification indicated that epigenetic factors (e.g., transcription regulatory modules) have influences on transcription that depend upon nucleotide sequences. Interestingly, the trend was not observed for |β|.

92% of *cis*-eQTLs were within their target genes or in 100 kb flanking regions, which is consistent with previous studies [3], [7]; and it was also consistent that most of large-effect eQTLs were located within 20 kb [29].

### 2) Multi-regulatory eQTLs

We identified 6 and 5 multi-regulatory *cis*- and *trans*-eQTLs, respectively. We note that a pair of multi-regulatory *cis*-eQTLs on chr12, rs2416549 and rs35969491, and another pair of multi-regulatory *trans*-eQTLs on chr3, rs11718621 and rs6773917, each are likely to indicate the same locus because they were each close (*r* ^2^ = 0.99 and 0.39, respectively) and the regulated gene sets are similar. Multi-regulatory eQTLs may comprise two types of eQTLs; some may be true master regulators, while others may each comprise a group of eQTLs in strong LD, each of which regulates one gene. Further studies are needed to identify more multi-regulatory eQTLs so that they would be further analyzed in terms of LD structure and effect sizes comparing with eQTLs regulating one gene. Presence of *trans-*acting master regulators has been increasingly suggested[30]–[32]. However, it is very challenging to identify master regulators because statistical power to detect *trans*-eQTLs is low because of multiple testing corrections. Interestingly, with the stringent threshold of this study, *trans*-regulated genes were often associated with multi-regulatory *trans*-eQTLs (Figure S5), which may suggest multi-regulatory *trans*-eQTLs tend to have large effects.

### 3) mRNA and lincRNA transcripts

The importance of lincRNAs to phenotypic variation is increasingly recognized; nevertheless, previous eQTL studies focused only on coding genes, and did not include analyses of lincRNA transcripts. Here, we examined the genetic causes of variation in expression of coding genes and of lincRNAs. Coding genes and lincRNAs exhibited different characteristics; for example, the proportion of *cis*-regulated transcripts was 3 times larger for mRNAs (15.1% vs. 4.8%, Table 1); sequence variations influence coding gens more than lincRNAs. Nevertheless, eQTLs for lincRNAs should not be ignored because still 5.3% of lincRNAs were regulated by either *cis-* or *trans-*eQTLs, and the mean *R* ^2^ values of *cis*- or *trans*-eQTLs regulating lincRNAs were as large as those regulating mRNAs (Table 1, Wilcoxon's rank-sum *P* = 0.094), and |β| values were even larger (Table 1, Wilcoxon's rank-sum *P* = 3.2E-14), which might indicate that lincRNAs are more variable than mRNAs, while the eQTL effects were similar in terms of *R* ^2^. These differences and similarities between coding transcripts and lincRNAs may indicate interesting mechanisms underlying the expressional regulations.

### 4) Application to GWAS results

The rationales behind utilizing eQTL mapping to interpret GWAS are that evidence from GWAS supports that transcriptional alterations contribute to risks of complex diseases; 1) a substantial fraction of GWAS-identified SNPs fell intergenic regions; and 2) eQTLs identified in previous study are enriched in GWAS-reported SNPs. Indeed, our eQTLs were also enriched in GWAS-reported SNPs: 1.7-fold for *cis*-eQTLs (one-sample proportion test *P*<2.2E-16) and 3.7-fold for *trans*-eQTLs (one sample proportion test *P* = 3.5E-15). Interestingly, *trans-*eQTLs were more enriched than *cis*-eQTLs. We identified 386 records for which our eQTL map may provide another evidence to interpret GWAS results. We emphasize that our results of applying our eQTL map to GWAS interpretation can only suggest another possibilities for candidate causative genes based on expressional variations and that the significant association with expression does not necessarily indicate the gene is causative (an example was shown for *RPS26* and type I diabetes [33]). Thorough and close assessment is required for each case to conclude what gene is truly causative. Still, reviewing previous GWAS results while referring to eQTL maps, not only regarding *cis*-eQTLs but also *trans-*eQTLs, would be worthwhile, and eQTL maps will provide useful information for interpreting and understanding future GWAS results as well.

### 5) Replication

*Cis*-regulated genes identified in our study were in a good concordance with those identified by Westra *et al*. [20]: 60.9% of our *cis*-regulated genes were replicated. The 60% replication rate seems reasonable for whole blood samples because, in the current study, we replicated 56% of 112 *cis*-regulated genes identified in a previous study [17] for whole blood samples from 76 Japanese individuals. On the other hand, replication of *trans*-eQTLs was challenging; only <1% of *trans*-eQTLs identified by Westra et al. [20] were replicated in the current study. Variation between different populations might be important for *trans*-eQTLs because we could replicate one of two *trans*-eQTLs in the previous study for the Japanese population [17]. We speculate the reason of low replication for *trans*-eQTLs as follows: Mechanisms of *trans-*effects of many sequence variations are considered as that a variant induces transcriptional alteration in a *cis* manner or functional change by substituting amino acids of proteins that involve in transcriptional regulation of other genes, and then, the locally induced change causes changes in expression levels of other genes [34]. Although *trans-*regulatory mechanisms are largely unknown, such a regulatory system may depend on a network of genes in which the genes interactively and cooperatively work in the same biological process; consequently, individual out-put gene expression levels are a cumulative result of a net effect of the whole network which could involve complex feedback mechanisms. The state of such a network should change dynamically with cell types, environmental conditions, and time. This is one of the reasons for the low reproducibility of *trans-*eQTLs. It should be noted that our *trans-*eQTLs were identified under just one set of conditions; therefore, the validity of applying our results to situations that represent different conditions needs to be carefully evaluated. However, we believe that our *trans*-eQTL analysis provides general insights into *trans-*effects, such as how effect magnitudes, β or *R* ^2^, are distributed.

## Methods

### Subjects and ethics statement

The study subjects were 301 apparently healthy individuals residing in Nagahama City, Japan. All participants provided written informed consent. The study protocol was approved by the Ethics Committee of Kyoto University Graduate School and Faculty of Medicine.

### SNP genotyping and quality control

We extracted DNA from leukocytes and carried out genome-wide SNP genotyping with the Infinum HumanOmni5Exome BeadChip (Illumina, Inc., San Diego, CA, USA). We excluded any SNP with a missing rate >1%, Hardy-Weinberg equilibrium test *P* value<1E-07, minor allele frequency <5%, or that mapped to a sex chromosome. Ultimately, we examined a final set of 1,425,832 autosomal SNPs in the analysis. We excluded three samples from the analysis; one was excluded because of unsuccessful DNA extraction, and two others were excluded because of kinship with other sample. The snpStats package (http://www.bioconductor.org/packages/release/bioc/html/snpStats.html) in Bioconductor [35] was used to conduct the principal component analysis, and no subjects were identified as outliers relative to the HapMap JPT (Figure S2).

### Gene expression profiles

Whole blood was collected from each participant when in a non-stimulated state; PAXgene Blood RNA Kits (QIAGEN, Hilden, Germany) were then used to collect samples of total RNA. For each participant, we used the Low Input Quick Amp Labeling Kit (Agilent Technologies, Inc., Santa Clara CA, USA) according to the manufacturer’s protocol and 100 ng of total RNA to synthesize each labeled cRNA sample. We used Gene Expression Hybridization kits (Agilent Technologies, Inc.) to hybridize labeled cRNA to arrays from SurePrint G3 Human Gene Expression 8×60 K Microarray Kits (Agilent Technologies, Inc., design ID: 028004); Gene Expression Wash Packs (Agilent Technologies, Inc.) were then used according to the manufacturer’s protocols to wash each microarray. Each microarray was scanned with a DNA Microarray Scanner (Agilent Technologies, Inc.), and Feature Extraction Ver.9.5.3 (Agilent Technologies, Inc.) was used to measure signal intensity.

### Normalization and exclusion of expression data

The data were processed using the GeneSpringGX11 as follows. For each set of duplicated probes, the mean signal intensity was calculated. Signal intensities less than 1 were each set to 1, and each signal intensity value was transformed by taking the binary logarithm. Normalization was carried out by a 75th percentile shift; this normalization procedure was recommended by Agilent. After this normalization, the 75th percentile signal intensity of each chip was set to 0, at which point the signal values ranged from –7.3 to 12.3 with a median (mean) of –2.6 (–2.1).

We excluded 5,550 probes for which we were not able to obtain specific positions on the chromosomes of their target genes and 1,488 probes that were mapped on the sex chromosomes. We did not filter any probes based on expression abundance because the information that the transcript is not expressed might be of biological importance. However, signal values for low or non-expressed genes are often unreliable; therefore, we show median expression values for our eQTL-regulated transcripts provided in File S1; and to interpret the expression values Figure S3 shows how expression values were distributed for expressed or non-expressed transcripts.

### Annotation of expression microarray probes

Annotation for gene expression probes of our chip (Agilent Technologies, Inc., design ID: 028004) was obtained from eArray (release date: 2012/04/11, build version: hg19:GRCh37:Feb2009, available online https://earray.chem.agilent.com/earray/). We defined three groups of probes: probes for *mRNA* transcripts, probes for *lincRNA* transcripts, and probes for *other* transcripts. Probes were classified into the *mRNA* group if they had assigned RefSeq NM accession numbers. *lincRNA* probes were indicated as such in Agilent’s annotation. All the other probes were classified into the *other* group. The transcription start and end sites of genes represented by the probes that were classified into *mRNA* or *other* were obtained from a seq_gene.md file downloaded from the NCBI website (http://www.ncbi.nlm.nih.gov/accessed on 2013/02/20); and those represented by *lincRNA* probes were obtained from either Agilent’s annotation or lincRNAsTranscripts table downloaded from the UCSC Genome Browser (http://genome.ucsc.edu/accessed on 2013/04/09).

### Annotation of SNPs

BLAST was used to map probes from the SNP genotyping array into GRCh37; a rsID was assigned to each SNP based on its mapped chromosomal position on GRCh37. We defined a distance between a SNP and a gene as base pairs between the chromosomal position of the SNP and the position of the nearest transcription start/end site of the gene. If the SNP was located within the gene, then the distance was set to 0. Directions of genes were considered, and the sign associated with each distance indicated that the SNP was located upstream (negative) or downstream (positive) of the gene. ANNOVAR version 2013-05-09 [36] (http://www.openbioinformatics.org/annovar/) was used to annotate SNPs for classification into gene-structure-based categories; the RefSeq Gene (build version 19) was used as the reference. We annotated SNPs with ANNOVAR’s default definitions and precedence of SNP functional categories if a SNP was located within its target gene or within 1 kb-flanking regions of its target gene, and the gene name in the ANOVVAR annotation matched the target gene (if the gene name did not match, no specific functions were assigned); and otherwise, the SNP was categorized into *intergenic* (see supplementary note in File S3 for details). Using this method, we would classify an eQTL as intergenic if it was located outside its target gene even though it was located within another gene; in a different example, an eQTL in an intron of its target gene was classified as intronic even though it was located in any other category of another gene.

We classified each intergenic SNP into one of the regulatory potential classes as defined based on epigenetic information available in public databases by RegulomeDB [19] for dbSNP132 (downloaded from http://regulome.stanford.edu/on 2013/07/24). We were able to assign a regulatory classification to each of 1,396,242 SNPs (97.9% of the tested SNPs). We considered seven categories (Category 1–7) of regulatory classes as defined by the RegulomeDB, but we did not use the 15 subcategories (1a–f, 2a–c, 3a–b, 4–7). Briefly, lower scores indicated more evidence for the SNP being located in a regulatory region. Each known eQTL with known additional epigenetic functional annotation was assigned to Category 1. Category 2 requires direct evidence of binding through ChIP-seq and DNase. Category 3 requires a less complete set of evidence of binding. Categories 4–6 each comprised SNPs with minimal evidence of effects on transcription factor binding; Category 4 SNPs had DNase and ChIP-seq evidence; Category 5 SNPs had DNase or ChIP-seq evidence; and Category 6 had any single annotation not categorized above. Finally, Category 7 SNPs had no known evidence of TF binding.

### eQTL mapping

We performed surrogate variable analysis [37] to identify unmodeled latent factors that cause heterogeneity in expression data. We identified two significant surrogate variables with age and gender used as known covariates using sva package (http://bioconductor.org/packages/release/bioc/html/sva.html) in Bioconductor [35], [38]. We corrected expressions of each transcript for age, gender, and the two surrogate variables by fitting a multiple linear model in R version 3.0.2 (http://www.R-project.org/). We further excluded 4,972 probes that were mapped to regions with SNPs that was found polymorphic in the HapMap JPT samples or our study subjects because polymorphisms in such regions can alter hybridization efficiency; consequently, signal intensities may not reflect the actual amount of RNA [39]–[42]. The remaining 30,395 probes were included in the analysis. We assumed an additive model for all SNPs, and we coded each SNP genotypes as 0, 1, or 2, to represent the number of minor alleles in each individual. PLINK v1.07 [43] (http://pngu.mgh.harvard.edu/purcell/plink/) was used to perform the association analysis between each adjusted transcriptional phenotype and each of 1,425,832 autosomal SNPs with 298 individuals.

We define a *local* SNP as a SNP located on the same chromosome and within 500 kb from the nearest transcription start/end site of the gene that encodes the transcript, and a *distant* SNP, otherwise. We defined a *cis*-eQTL as a local SNP that significantly affects expression of a gene; similarly we defined a *trans*-eQTL as a distant SNP that significantly affects expression of a gene. We examined 16,986,695 local SNP-transcript pairs (11,028,260 for mRNAs, 3,485,407 for lincRNAs, and 2,473,028 for other transcripts). The mean number of local SNPs per probe was 560 (minimum 1, maximum 4,630). We examined about 43 billion distant SNP-transcript pairs. To identify *cis*-eQTLs, we estimated FDR with the permutation approach as described by Westra *et al.* [20]. Briefly, sample identifiers were permuted for 10 times, and only the local SNP with the smallest *P* value for each transcript was used to simulate the null distribution. With this approach we estimated FDR only for the SNP with the smallest *P* value for each transcript, and local SNPs with the FDR smaller than 5% were identified as *cis*-eQTLs. Therefore, no more than one *cis*-eQTL was identified for each transcript. If multiple SNPs in perfect LD (*r* ^2^ = 1) were the most significant with the same *P* value, the middle SNP was used to represent the eQTL. To exclude possible false discoveries caused by outliers or violation of normality assumptions, we performed Kruskal-Wallis test [44], a non-parametric test, and excluded *cis*-eQTL-transcript pairs with *P* value>0.00015 (see supplementary note in File S3).

To identify *trans*-eQTLs, we used the Bonferroni correction for multiple comparisons among the approximately 43 billion tests; only distant SNPs with nominal *P* values smaller than 1.15E-12, which corresponds to a family-wise error rate of 5%, were considered significant. We applied intensive exclusion criteria to obtain reliable *trans*-eQTLs. First, we excluded *trans-*eQTLs that may only capture *cis*-effects because of LD by a conditional regression on *cis*-eQTL genotypes (i.e., excluded when residuals of fitting *cis*-eQTL genotypes were not significantly associated with *trans*-eQTL genotypes by *P*<0.05). This analysis was performed when a *trans-*eQTL and its target transcript were located on the same chromosome, and a *cis*-eQTL was also identified for the transcript (*cis*-eQTLs excluded by Kruskal-Wallis tests were also considered). Second, we excluded redundant *trans*-eQTLs because of LD with other *trans*-eQTLs by sequential conditional regressions. For each transcript, *trans-*eQTLs on the same chromosome were iteratively tested starting from the *trans*-eQTL of the smallest *P* value for the transcript. If significant (*P*<0.05), the *trans*-eQTL is kept and residuals were used for the next iteration. If not, the *trans*-eQTL was excluded as redundant, and residuals were not taken for the next iteration. After this procedure, we tested *trans-*eQTLs that were found significant in the sequential conditional regression all together with a multiple linear regression, and non-significant *trans-*eQTLs (*P*>0.05) were further removed. Third, in order to confirm that the *trans-*eQTLs were not false positives because of cross-hybridization of probes to unexpected transcripts near the *trans-*eQTLs, we mapped the probe sequence to the flanking region (±500 kb) of its *trans-*eQTL by SHRiMP v.2.2.3 [45] for each probe-*trans-*eQTL combination. The human reference DNA sequence (GRCh37.p5) was downloaded from the NCBI (http://www.ncbi.nlm.nih.gov/). We used the same relaxed settings as Westra *et al*. [20] (match score of 10, mismatch score of 0, gap open penalty of −250, gap extension penalty of −100, and minimal Smith-Waterman score of 30%); -m 10 -i 0 -q -250 -f -100 -h 30%. We excluded a *trans-*eQTL if its associated probe was mapped to its flanking region. Fourth, we excluded low expression transcripts whose median expression levels were lower than −4.5 because we observed deviation from the distribution of median expression levels of *cis*-regulated transcripts (Figure S6). The cutoff was defined as the 5th percentile of the median expression levels of the *cis*-regulated transcripts. Kruskal-Wallis tests for the remaining SNP-transcript pairs were all significant (*P*<0.00015). We used the remaining *trans*-eQTLs in the further analyses.

The approach we used to correct for multiple testing with local SNPs differed from that used with distant SNPs because the high peak at low *P* values observed with local SNPs indicated that a substantial fraction of local SNPs were truly associated with the expression phenotype of one or more transcripts, whereas the uniform distribution of *P* values observed with distant SNPs indicated that the null hypothesis was true for most of the tests (Figure S1).

### Identifying multi-regulatory eQTLs

We defined a multi-regulatory *cis*-eQTL as a *cis*-eQTL that is associated with expression levels of at least three different local protein-coding genes (assigned RefSeq NM accessions). For this, we did not count probes that cross-hybridize to other local genes associated with the same *cis*-eQTL by mapping probe sequences to the exon sequences with SHRiMP v.2.2.3 [45] using the same set of options used for detecting cross-hybridization for *trans-*eQTLs above.

Similarly, we defined a multi-regulatory *trans-*eQTL as a *trans-*eQTL that is associated with expression levels of at least 3 different distant protein-coding genes, after excluding cross-hybridized probes in the same procedure as used for multi-regulatory *cis*-eQTLs.

### Statistical analysis for eQTLs

For comparison of mean effects of gene-based functional categories, we excluded SNPs that we were not able to assign to a specific category; we also excluded categories that comprised fewer than 5 eQTLs. Values of |β| and *R* ^2^ were log-transformed and then subjected to the ANOVA; the ANOVA was followed by Tukey’s HSD test (which performs all pairwise comparisons between two subcategories for multiple testing correction). The trend of log-transformed |β| and *R* ^2^ values with seven RegulomeDB classes (Classes 1a–f, 2a–c and 3a–b were grouped as 1, 2 and 3, respectively) was tested with Jonckheere-Terpstra permutation test (one-sided, 100,000 permutations) provided in clinfun package (http://cran.r-project.org/web/packages/clinfun/index.html) in R version 3.0.2 (http://www.R-project.org/). *r* ^2^ of LD between SNPs were computed with PLINK v1.07 [43].

### Replication analysis

We downloaded the eQTL map by Westra *et al*. [20] from their browser (http://genenetwork.nl/bloodeqtlbrowser/), and annotation files for HT12v3 and Agilent Human Genome 4×44 K array from the GEO (http://www.ncbi.nlm.nih.gov/geo/). We matched Entrez GeneIDs to compare with the replication studies. We referred to GWAS catalog to obtain SNPs tested for *trans*-eQTL in [20] (SNPs reported by 16, July, 2011). We found 978 distant SNP-transcript pairs in our study that corresponded to rs IDs and Entrez Gene IDs tested in [20].

### Matching eQTLs with GWAS-identified SNPs

We downloaded 16,541 public GWAS records from the NHRGI GWAS Catalog (http://www.genome.gov/gwastudies/ accessed on 2014/04/23). We excluded 323 records with reported *P* values that were not significant (reported as NS or Pending); we excluded another 6,142 records because the reported SNPs were not included in our tested SNPs. Ultimately, we examined 10,076 records for 8,069 unique SNPs reported by 1,436 GWAS. We matched GWAS-reported SNPs to our eQTLs when they exactly matched or were in LD (*r* ^2^>0.8). We excluded records if conditional regression on genotypes of a GWAS-identified SNP was significant *P*<0.05 (File S2) because they might be false discoveries of trait-eQTL association where eQTL and GWAS-identified SNP are two different genetic factors [33]. When matching gene symbols, we also searched their aliases downloaded from the HGNC BioMart version 0.7 (http://www.genenames.org/biomart/ accessed on 2013/09/28).

### Accession numbers

Our expression microarray data are available at the NCBI’s Gene Expression Omnibus under accession number GSE53351.

## Supporting Information

Figure S1 **Histogram of** ***P*** **values of all association tests.** A) Histogram of *P* values obtained from the 16,986,695 association tests between all autosomal transcripts and local SNPs. The excess of smaller *P* values indicates that a substantial fraction of associations are truly positive. B) Histogram of *P* values obtained from about 43 billion association tests between all autosomal transcripts and distant SNPs. The almost uniformly distributed *P* values suggests that most of distant SNPs have no effects on transcriptional regulation, though a slight increase at the low *P* values in frequency indicates a tiny fraction of distant SNPs are truly positive. Also see File S3 for a comment about influence of surrogate variable analysis on the distribution.
(TIF)

Figure S2 **Principal component analysis of study population in comparison with HapMap samples.** The first and second principal components are shown. CEU: Utah residents with Northern and Western European ancestry from the CEPH collection; YRI: Yoruba in Ibadan, Nigeria; JPT: Japanese in Tokyo; CHB: Han Chinese in Beijing, China; Sample: samples of the current study.
(TIF)

Figure S3 **Distribution of normalized expression data.** Distribution of normalized expression data for all 42,405 probes and 298 samples are shown. “A” (absent) if a foreground signal is <2.6 SD of background signal; “M” (marginal) if it was saturated, not uniform in a spot, or not uniform among replicated probes, or “P” (present) otherwise. The number following each class name is the number of data classified into the class.
(TIF)

Figure S4 **Regression coefficients of multi-regulatory eQTLs.** Regression coefficients, β, of each multi-regulatory *cis*-eQTLs (A) or *trans*-eQTLs (B) are shown. Directions of effects of each multi-regulatory eQTL are consistent.
(TIF)

Figure S5 ***Trans*** **-eQTL map.** A) Chromosomal positions of *trans*-eQTLs are plotted against chromosomal positions of associated transcripts. B) –log_10_ *P* values of *trans*-eQTLs are plotted against the respective chromosomal positions. (C) –log_10_ *P* values of *trans*-eQTLs are plotted against the chromosomal positions of associated transcripts. The horizontal and vertical dashed lines separate chromosomes; the diagonal dashed line indicates that the *trans*-eQTL is located at the same chromosomal positions as transcripts. mRNA transcripts are shown in red; lincRNA transcripts are shown in green; and other transcripts are shown in black. –log_10_ *P* values are truncated at 50, and a triangle indicate truncation.
(TIF)

Figure S6 **Median expression levels of** ***cis*** **-regulated or** ***trans*** **-regulated genes.**
(TIF)

Table S1 **Demographic characteristics of study subjects.**
(DOCX)

Table S2 ***P*** **values of Tukey's HSD test.**
(DOCX)

Table S3 **Replicated** ***trans*** **-eQTLs identified by Westra** ***et al*** **.** [20].
(XLSX)

File S1 **Annotations and statistics of** ***cis*** **-eQTLs and** ***trans*** **-eQTLs.**
(XLS)

File S2 **Results of our application of eQTL map to GWAS records.** Sheet “Case1–3” shows records classified into Case 1, 2, or 3; sheet “Case 4” shows records classified into Case 4; sheet “Excluded” shows excluded records because GWAS SNP and eQTL are not likely to colocalize.
(XLSX)

File S3 **Supplementary notes.**
(PDF)
