# Supplementary figures and images for: Large-Scale East-Asian eQTL Mapping Reveals Novel Candidate Genes for LD Mapping and the Genomic Landscape of Transcriptional Effects of Sequence Variants
Source: PLoS One. 2014 Jun 23;9(6):e100924. doi: 10.1371/journal.pone.0100924 (PMC4067418; doi:10.1371/journal.pone.0100924)

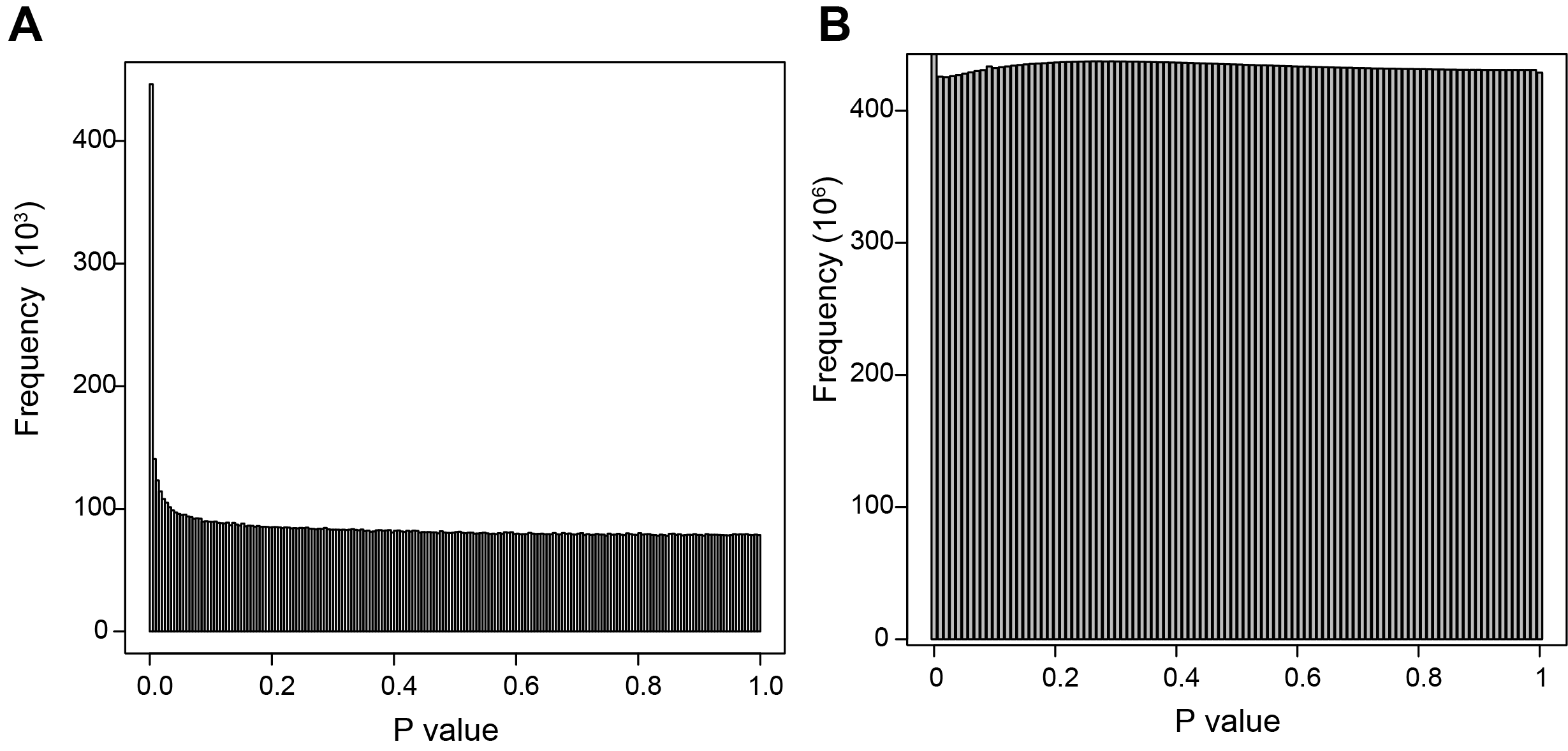

Supplement: Figure S1 — Histogram of P values of all association tests. A) Histogram of P values obtained from the 16,986,695 association tests between all autosomal transcripts and local SNPs. The excess of smaller P values indicates that a substantial fraction of associations are truly positive. B) Histogram of P values obtained from about 43 billion association tests between all autosomal transcripts and distant SNPs. The almost uniformly distributed P values suggests that most of distant SNPs have no effects on transcriptional regulation, though a slight increase at the low P values in frequency indicates a tiny fraction of distant SNPs are truly positive. Also see File S3 for a comment about influence of surrogate variable analysis on the distribution. (TIF) [file pone.0100924.s001.tif]

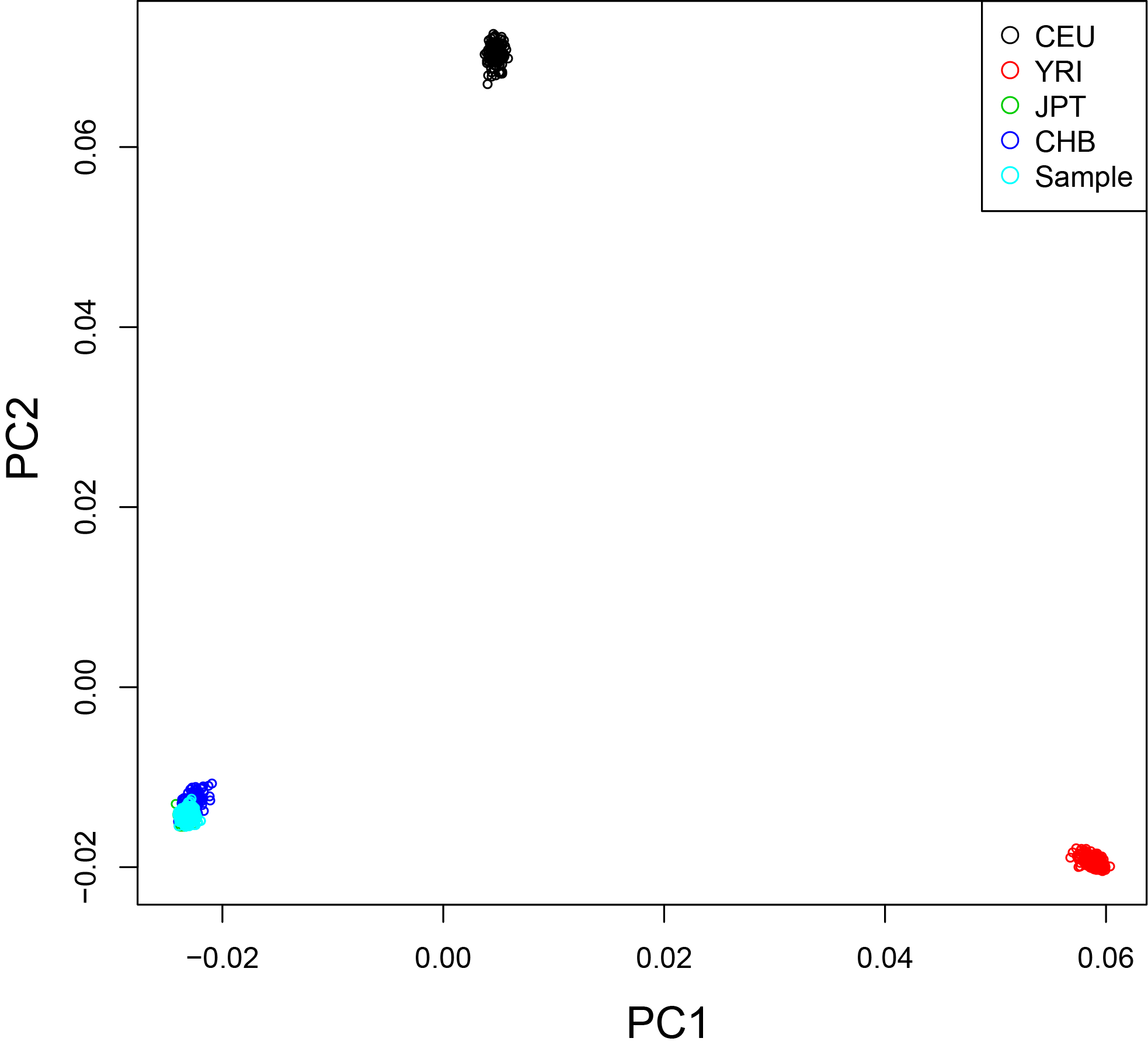

Supplement: Figure S2 — Principal component analysis of study population in comparison with HapMap samples. The first and second principal components are shown. CEU: Utah residents with Northern and Western European ancestry from the CEPH collection; YRI: Yoruba in Ibadan, Nigeria; JPT: Japanese in Tokyo; CHB: Han Chinese in Beijing, China; Sample: samples of the current study. (TIF) [file pone.0100924.s002.tif]

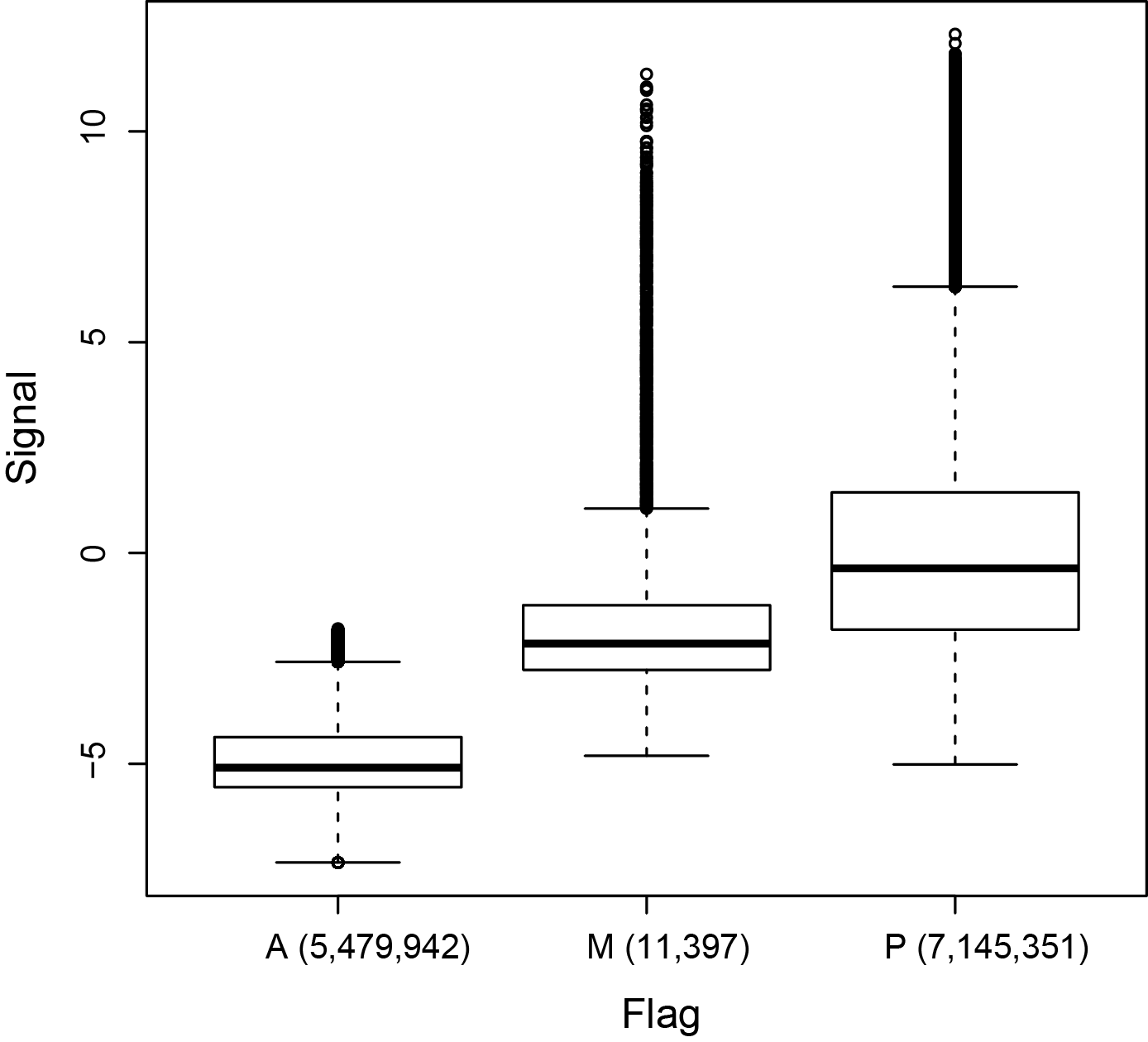

Supplement: Figure S3 — Distribution of normalized expression data. Distribution of normalized expression data for all 42,405 probes and 298 samples are shown. “A” (absent) if a foreground signal is <2.6 SD of background signal; “M” (marginal) if it was saturated, not uniform in a spot, or not uniform among replicated probes, or “P” (present) otherwise. The number following each class name is the number of data classified into the class. (TIF) [file pone.0100924.s003.tif]

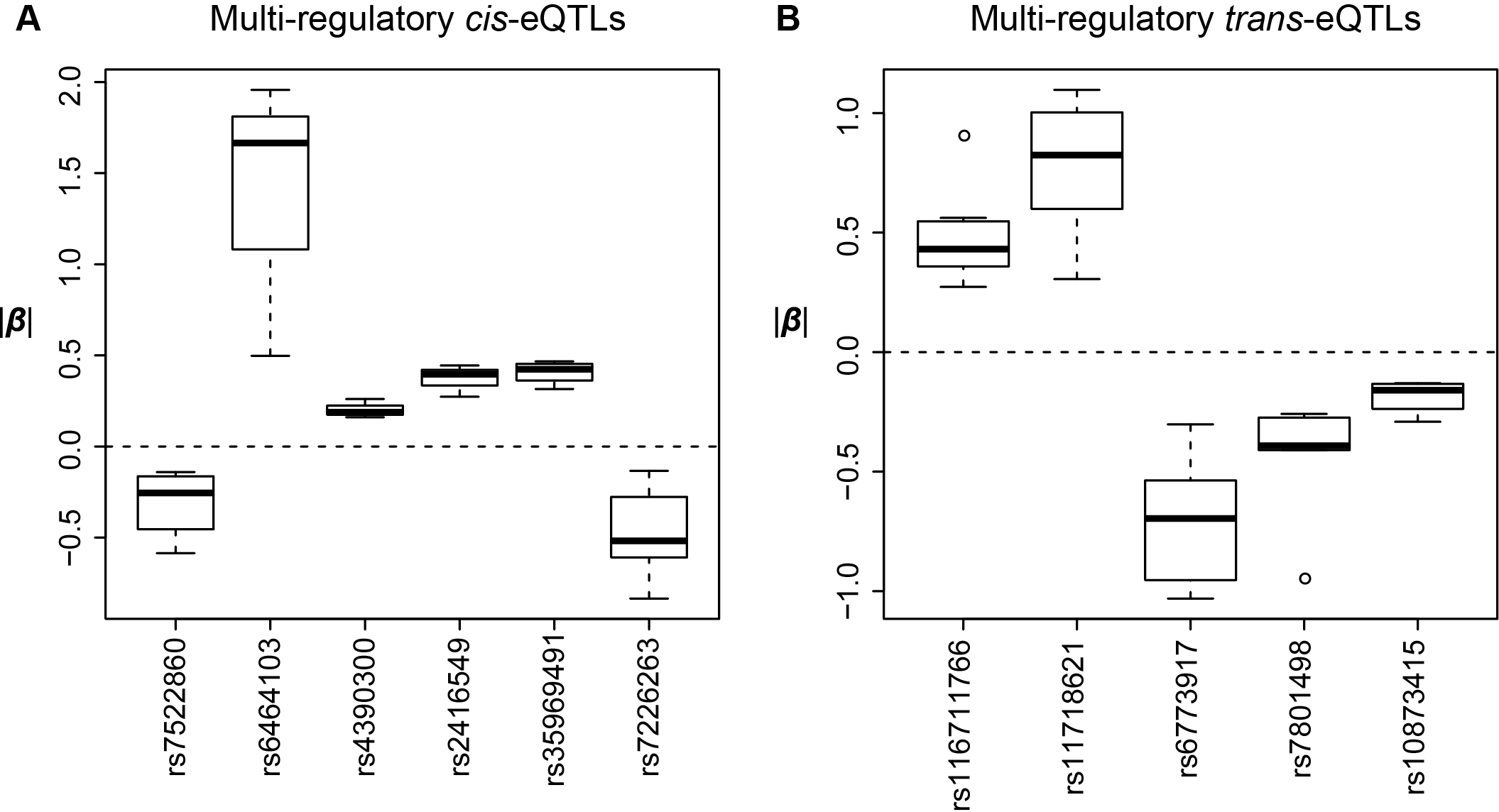

Supplement: Figure S4 — Regression coefficients of multi-regulatory eQTLs. Regression coefficients, β, of each multi-regulatory cis-eQTLs (A) or trans-eQTLs (B) are shown. Directions of effects of each multi-regulatory eQTL are consistent. (TIF) [file pone.0100924.s004.tif]

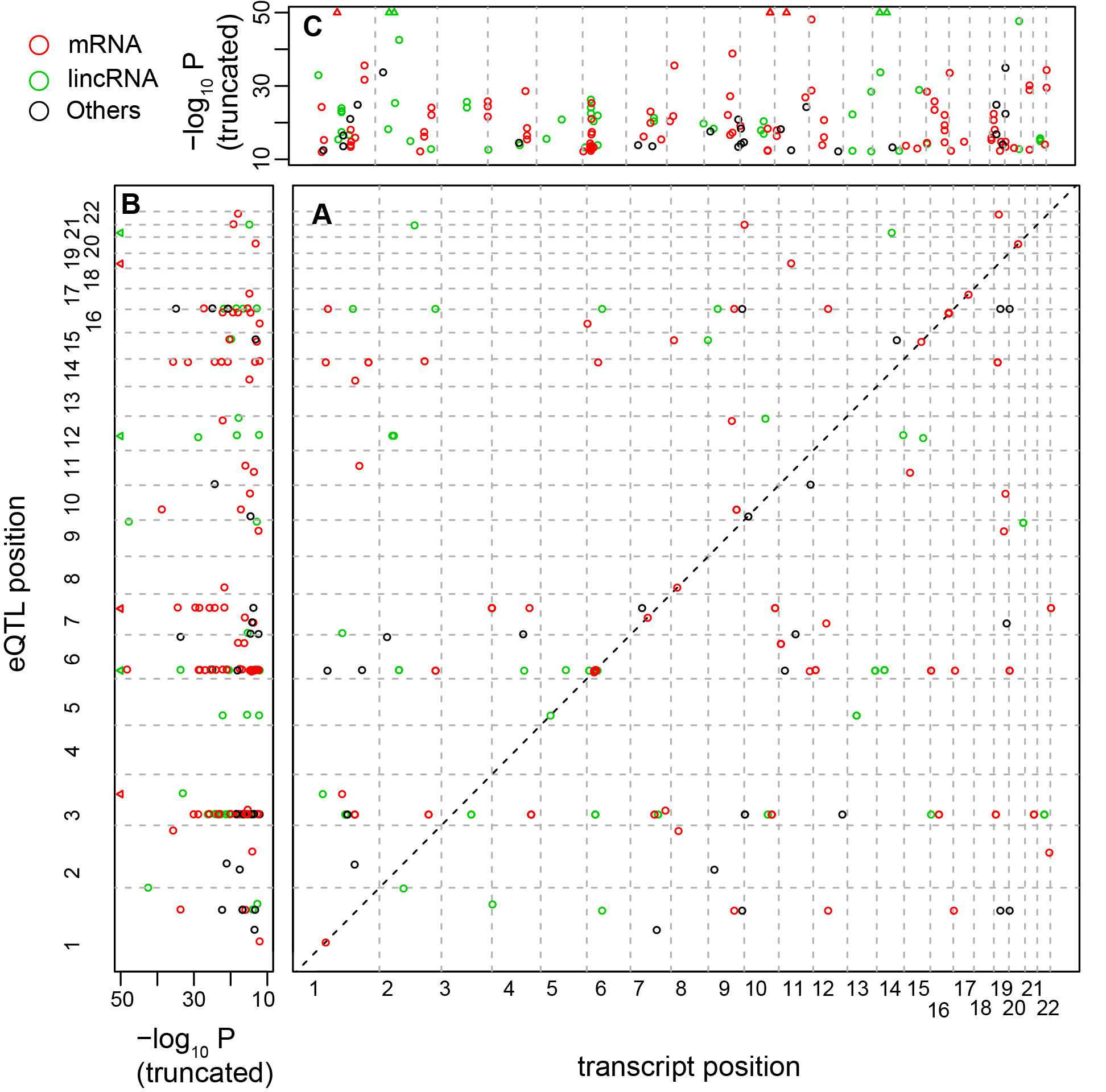

Supplement: Figure S5 — Trans -eQTL map. A) Chromosomal positions of trans-eQTLs are plotted against chromosomal positions of associated transcripts. B) –log10 P values of trans-eQTLs are plotted against the respective chromosomal positions. (C) –log10 P values of trans-eQTLs are plotted against the chromosomal positions of associated transcripts. The horizontal and vertical dashed lines separate chromosomes; the diagonal dashed line indicates that the trans-eQTL is located at the same chromosomal positions as transcripts. mRNA transcripts are shown in red; lincRNA transcripts are shown in green; and other transcripts are shown in black. –log10 P values are truncated at 50, and a triangle indicate truncation. (TIF) [file pone.0100924.s005.tif]

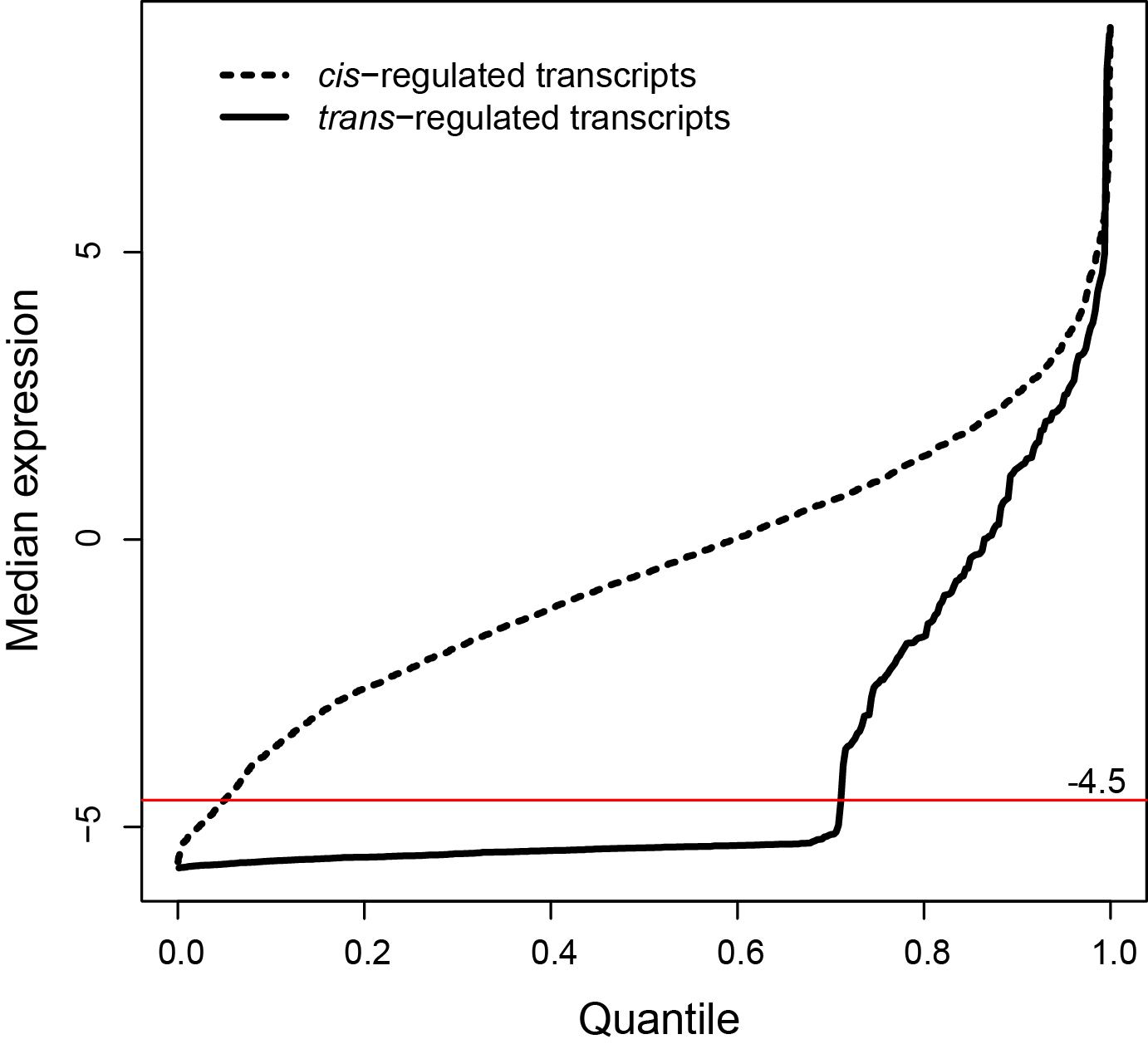

Supplement: Figure S6 — Median expression levels of cis -regulated or trans -regulated genes. (TIF) [file pone.0100924.s006.tif]
