# Supplementary material for: Large-Scale East-Asian eQTL Mapping Reveals Novel Candidate Genes for LD Mapping and the Genomic Landscape of Transcriptional Effects of Sequence Variants
Source: PLoS One. 2014 Jun 23;9(6):e100924. doi: 10.1371/journal.pone.0100924 (PMC4067418; doi:10.1371/journal.pone.0100924)
Supplement: Table S1 — Demographic characteristics of study subjects. (DOCX) [file pone.0100924.s007.docx]

**Table S1: Demographic characteristics of study subjects**

|  | All (n=298) | | Men (n=102) | | Women (n=196) | |
| --- | --- | --- | --- | --- | --- | --- |
|  | mean (SD) | min.-max. | mean (SD) | min.-max. | mean (SD) | min.-max. |
| Age | 55.1 (9.3) | 32 - 66 | 57.0 (8.8) | 32 - 66 | 54.1 (9.4) | 32 - 66 |
| Height (*cm*) | 161.1 (8.5) | 131.3-189.6 | 169.5 (6.6) | 154.2-189.6 | 156.7 (5.6) | 131.3-172.4 |
| Weight (*kg*) | 59.7 (10.3) | 40.3 - 90.3 | 67.6 (9.1) | 46.0 - 90.3 | 55.6 (8.4) | 40.3 - 85.4 |
| BMI (*kg*/*m*^2^) | 22.9 (3.1) | 16.5 - 34.6 | 23.5 (2.8) | 16.6 - 32.4 | 22.6 (3.2) | 16.5 - 34.6 |

SD: standard deviation; min.: minimum; max.: maximum; BMI: body mass index
