# Supplementary material for: Large-Scale East-Asian eQTL Mapping Reveals Novel Candidate Genes for LD Mapping and the Genomic Landscape of Transcriptional Effects of Sequence Variants
Source: PLoS One. 2014 Jun 23;9(6):e100924. doi: 10.1371/journal.pone.0100924 (PMC4067418; doi:10.1371/journal.pone.0100924)
Supplement: Table S2 — P values of Tukey's HSD test. (DOCX) [file pone.0100924.s008.docx]

**Table S2: P values of Tukey's HSD test**

|  | Exonic | Intronic | 3’ UTR | 5’ UTR | Upstream | Downstream | Intergenic |
| --- | --- | --- | --- | --- | --- | --- | --- |
| Exonic |  |  |  |  |  |  |  |
| Intronic |  |  |  |  | 0.016 |  | 0.026 |
| 3’ UTR |  |  |  |  | 2.0E-04 |  |  |
| 5’ UTR |  |  |  |  |  |  |  |
| Upstream |  |  |  |  |  | 0.033 | 8.8E-05 |
| Downstream |  |  |  |  |  |  |  |
| Intergenic |  | 4.0E-07 | 7.5E-04 |  | 0.034 |  |  |

P values obtained from comparison of |β| and *R*^2^ are shown in the upper and lower triangles, respectively. Empty cells indicate that the comparison was not statistically significant by the corrected P value of 0.05.
