# Supplementary material for: Large-Scale East-Asian eQTL Mapping Reveals Novel Candidate Genes for LD Mapping and the Genomic Landscape of Transcriptional Effects of Sequence Variants
Source: PLoS One. 2014 Jun 23;9(6):e100924. doi: 10.1371/journal.pone.0100924 (PMC4067418; doi:10.1371/journal.pone.0100924)
Supplement: File S3 — Supplementary notes. (PDF) [file pone.0100924.s012.pdf]

Large-scale East-Asian eQTL Mapping Reveals Novel Candidate Genes for LD Mapping and the Genomic Landscape of Transcriptional Effects of Sequence Variants

Maiko Narahara,[1] Koichiro Higasa,[2] Seiji Nakamura,[3] Yasuharu Tabara,[2] Takahisa Kawaguchi,[2]

Miho Ishii,[3] Kenichi Matsubara,[3] Fumihiko Matsuda,[2] Ryo Yamada[1]*

[1]Statistical Genetics, Center for Genomic Medicine, Kyoto University Graduate School of Medicine, Kyoto, Japan
[2]Human Disease Genomics, Center for Genomic Medicine, Kyoto University Graduate School of Medicine, Kyoto, Japan
[3]DNA Chip Research Inc., Kanagawa, Japan
*Correspondence: ryamada@genome.med.kyoto-u.ac.jp

Supplementary note

β and R^2 as indices for effect magnitudes

β, or |β|, is the coefficient (or the absolute value of the coefficient) of genotypes for an eQTL, and represents the effect size of a minor allele for means; specifically, how much the mean expression level is changed by possessing one minor allele on a $\log_2$ scale (i.e., β=1 means that the expression levels double per minor allele). R^2 is the proportion of the regression sum of squares to the total sum of squares, and this proportion represents the proportion of phenotypic variance explained by genotype: i.e., R^2 represents how well the genotypes of a SNP explain the variance in an expression phenotype. Because we did not scale expression phenotypes by the standard deviation, β can be correlated to variability of the phenotype, while R^2 is not influenced by variability. We showed results for both measures because β and R^2 are two different measures for effects of predictor variables. When two eQTLs have the same R^2 values but different β values, the proportion of variance explained by genotypes is the same but the difference in means between two genotypes, say genotypes AA and Aa, is different. When two eQTLs have the same β values but different R^2 values, the difference in means between two genotypes, say genotypes AA and Aa, is the same, but the proportion of explained variance is different. β is important because it represents effect sizes, not statistical significance. R^2 is closely related to *P* values; with the same sample sizes, comparing R^2 is equivalent to comparing *P* values. R^2 is also important because it represents the narrow-sense heritability, h^2, where the SNP is the only genetic factor for the phenotype. If there are more than one independent genetic factors, h^2 is given by the sum of R^2 of all the genetic factors.

In our study, many results showed substantial discordance between β and R^2. For example, genic and intergenic *cis*-eQTLs were different in R^2 but not obviously different in β (Figure 3A and 3B); association with RegolumeDB was significant for R^2 but not for β (Figure 4); relationship between R^2 or β and eQTL-gene distance was also different (Figure 5C and 5F). Regarding these differences, we consider that R^2 is the better index to represent eQTL effects because R^2 was more consistent with known biological evidences, and also because β is influenced by variability of phenotype.

However, as mentioned above, β indicates how an eQTL can change the mean expression levels, which might be of greater interest than statistical significance represented by R^2. Therefore, we showed results for both β and R^2.

Definitions of Case 1-4 for GWAS results

We defined the following four cases for GWAS results in which *cis* or *trans* effects were found for reported SNPs. A GWAS result is classified into Case 1 when the GWAS-suggested causative gene differs from the gene regulated by the GWAS-identified SNP or when GWAS could not suggest any gene because the GWAS-identified SNP fell the intergenic region; Case 2 when expressions of the GWAS-reported gene is regulated by the GWAS-identified SNP (i.e., our eQTL map supports the involvement of the gene); Case 3 when the eQTL map helped to prioritize multiple genes inconclusively reported by the GWAS; or Case 4 when a GWAS-identified SNP influences expression of a gene on a different chromosome without *cis*-effects. Here, we considered the GWAS-identified SNP is a *trans*-eQTL without *cis*-effects when the following three conditions were satisfied: 1) the GWAS-identified SNP was a *trans*-eQTL (or in LD with a *trans*-eQTL) for a gene on the different chromosome, 2) no SNPs in LD ($r^2 > 0.8$) with the GWAS-identified SNP were *cis*-eQTLs; 3) the *trans*-eQTLs and the most significant local SNP for their target genes were unlinked ($r^2 < 0.009$) to confirm that the *trans*-eQTLs were truly on the different chromosome. The threshold for unlinked SNPs was determined based on the distribution of r^2 of all pairs of SNPs on different chromosomes generated using randomly sampled 5,000 SNPs from our tested SNPs; and we chose the 90th percentile as a cutoff.

Surrogate variable correction and distribution of P values for all distant SNPs

The surrogate variable analysis (SVA) identifies unmodeled latent factors that cause heterogeneity in expression data [1]. We identified two significant surrogate variables (SV), and we corrected each expressional phenotype for age, gender, and the two SV. It was shown that SVA improved eQTL reproducibility [2]. In our data, we identified more *trans*-eQTLs with SV correction than without SV correction (we did not check whether or not more *cis*-eQTLs are found with SV

correction). Therefore, we consider that SVA improves eQTL identification. We note, however, that adding SV correction to age and gender adjustment changed the distribution of P values of all distant SNPs. As shown in Figure SN1, with SV correction, the distribution of P values became conservative, with disregarding enrichment of small P values, than expected distribution from complete null hypotheses.

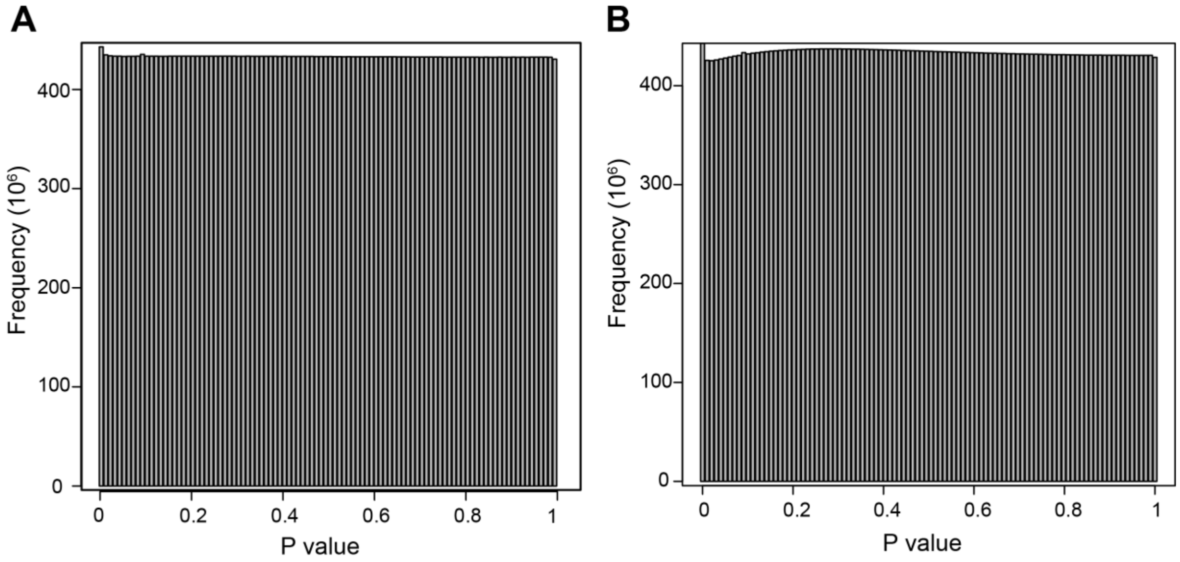

Figure SN1. Distribution of *P* values for all distant SNP-probe association tests. A) Without SV correction, B) with SV correction

Gene structure-based functional classification of SNPs

We annotated with ANNOVAR's default definitions and precedence of SNP functional categories (the numbers represent the precedence): *exonic(1)*: variants overlaps a coding exon; *splicing(1)*: variant is within 2-bp of a splicing junction in an intron; ncRNA(2): variant overlaps a transcript without coding annotation in the gene definition; 5' *UTR(3)*: variant overlaps a 5' untranslated region; 3' *UTR(3)*: variant overlaps a 3' untranslated region; *intronic(4)*: variant overlaps an intron; *upstream*(5): variant overlaps 1-kb region upstream of transcription start site; *downstream*(5): variant overlaps 1-kb region downstream of transcription end site; *intergenic*(6): variant is in intergenic region. Functions only available from ncRNA databases were also included. We also used ANNOVAR's default definitions of exonic functional categories in order of precedence as follows: *stopgain*, variant that leads to the immediate creation of stop codon at the variant site;

stoploss, variant that leads to the immediate elimination of stop codon at the variant site; *nonsynonymous* SNV, a single nucleotide change that cause an amino acid change; *synonymous* SNV, a single nucleotide change that does not cause an amino acid change. Functional changes caused by indels were not shown here because no SNP was assigned to these categories.

Exclusion of possible false eQTLs caused by outliers or violation of normality assumption

To exclude possible false discoveries caused by outliers or violation of normality assumption made for a linear regression, non-parametric tests or inverse normal transformation is commonly used. We considered applying either method to assure that our eQTLs are not false discoveries caused by such reasons. To employ more stringent method for our data, we evaluated the two methods; 1) Kruskal-Wallis test [3], and 2) linear regression following rank-based inverse normal transformation [4] (INT+LR). We tested pairs of the most significant local SNP and transcript with each method (Figure SN2), and compared with a linear regression (LR), which was performed as described in Methods (Figure SN2). The significance thresholds for LR was determined by the permutation FDR (as described in Methods), and those for Kruskal-Wallis test and INT+LR were determined based on a receiver operating characteristic (ROC) curve analysis [5] (the closest point to the upper-left corner) using the significance by LR as a golden standard. We identified 200 and 155 possible false positives with Kruskal-Wallis test ($P < 0.00015$) and INT+LR ($P<2E-05$), respectively (those in the lower-right region in Figure SN2). Therefore, we employed Kruskal-Wallis test, which gave more stringent criteria.

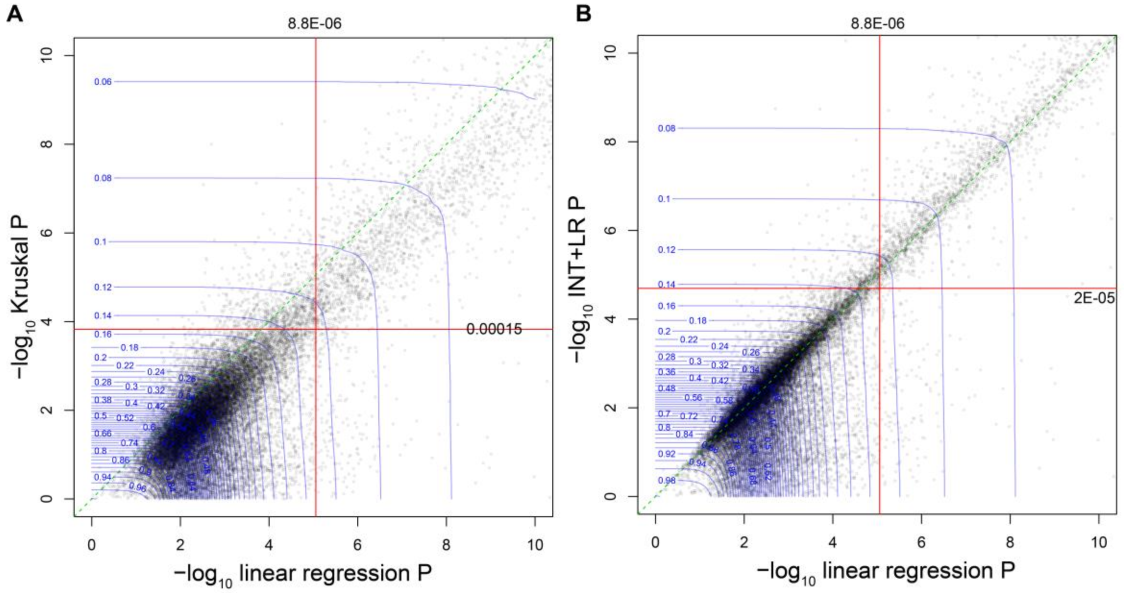

Figure SN2. Detection of possible false positives due to outliers or violation of normality assumption. *P* values with Kruskal-Wallis test (A) or linear regression following rank-based inverse normal transformation (INT+LR) (B) are plotted against *P* values with linear regression (LR). X and Y axes are truncated at 10. The red lines indicate significance threshold for each method. The contour lines indicate proportion of tests that were significant with both methods with respective cutoffs.

References

1. Leek JT, Storey JD (2007) Capturing heterogeneity in gene expression studies by surrogate variable analysis. PLoS Genet 3: 1724–1735.

2. Innocenti F, Cooper GM, Stanaway IB, Gamazon ER, Smith JD, et al. (2011) Identification, replication, and functional fine-mapping of expression quantitative trait loci in primary human liver tissue. PLoS Genet 7: e1002078.

3. Kruskal WH, Wallis WA (1952) Use of Ranks in One-Criterion Variance Analysis. J Am Stat Assoc 47: 583–621.

4. Beasley TM, Erickson S, Allison DB (2009) Rank-based inverse normal transformations are increasingly used, but are they merited? Behav Genet 39: 580–595.

5. Swets JA (1988) Measuring the accuracy of diagnostic systems. Science 240: 1285–1293.
